# Time Regained: When People Stop a Physical Activity Program, How Does Their Time Use Change? A Randomised Controlled Trial

**DOI:** 10.1371/journal.pone.0126665

**Published:** 2015-05-29

**Authors:** Sjaan Gomersall, Carol Maher, Coralie English, Alex Rowlands, Tim Olds

**Affiliations:** 1 Alliance for Research in Exercise, Nutrition and Physical Activity (ARENA), Sansom Institute for Health Research, University of South Australia, Adelaide, Australia; 2 Centre for Research on Exercise, Physical Activity and Health, School of Human Movement and Nutrition Sciences, The University of Queensland, Brisbane, Australia; 3 International Centre for Allied Health Evidence, Sansom Institute for Health Research, University of South Australia, Adelaide, Australia; Québec Heart and Lung Institute, CANADA

## Abstract

The aim of this study was to investigate how previously inactive adults who had participated in a structured, partly supervised 6-week exercise program restructured their time budgets when the program ended. Using a randomised controlled trial design, 129 previously inactive adults were recruited and randomly allocated to one of three groups: a Moderate or Extensive six-week physical activity intervention (150 and 300 additional minutes of exercise per week, respectively) or a Control group. Additional physical activity was accumulated through both group and individual exercise sessions with a wide range of activities. Use of time and time spent in energy expenditure zones was measured using a computerised 24-h self-report recall instrument, the Multimedia Activity Recall for Children and Adults, and accelerometry at baseline, mid- and end-program and at 3- and 6-months follow up. At final follow up, all significant changes in time use domains had returned to within 20 minutes of baseline levels (Physical Activity 1-2 min/d, Active Transport 3-9 min/d, Self-Care 0-2 min/d, Television/Videogames 13-18 min/d in the Moderate and Extensive group, relative to Controls, respectively, p>0.05). Similarly, all significant changes in time spent in the moderate energy expenditure zone had returned to within 1-3 min/d baseline levels (p>0.05), however time spent in vigorous physical activity according to accelerometry estimates remained elevated, although the changes were small in magnitude (1 min/d in the Moderate and Extensive groups, relative to Controls, p=0.01). The results of this study demonstrate strong recidivist patterns in physical activity, but also in other aspects of time use. In designing and determining the effectiveness of exercise interventions, future studies would benefit from considering the whole profile of time use, rather than focusing on individual activities.

**Trial Registration:**

Australian New Zealand Clinical Trials Registry ACTRN12610000248066

## Introduction

When people start an exercise program, the time needed for exercise, changing in and out of exercise clothing, showering, and getting to and from the exercise venue must be drawn from other “time reservoirs”. Our previous work has shown that this time is largely drawn television viewing [1]. This study addresses a related question: when people *stop* a structured, externally imposed exercise program, what happens to the time that has been “freed up”? How do people restructure their time budgets? Is extra time devoted to similar or alternative forms of physical activity? Are there enduring changes in time use, or is the pre-program pattern of time use resumed?

The level of recidivism in physical activity programs is notoriously high, often cited at 50% drop-out after 6 months [2], even when the exercise stimulus (i.e. the structured program) continues. However, follow-up measures using typical physical activity questionnaires may be misleading, because former participants may adopt other types of physical activity after the exercise stimulus has stopped, such as gardening or walking, which may not be captured by the questionnaires [3, 4]. Questionnaires may also fail to capture shifts from sedentary behaviours to light physical activity.

When individuals cease a structured physical activity program, the time previously committed to the program is freed up. How this time is redistributed can have important health consequences. If the liberated time is devoted to television viewing, for example, then there may be a negative effect on health [5, 6]. If it is devoted to sleep, the detrimental effects may be mitigated, since poor sleep has been associated with greater risk of obesity [7] and depression [8]. It is possible that participation in a structured program may “reset” a hypothetical “physical activity setpoint” [9]. This may lead to time freed up from a structured exercise program being devoted to alternative physical activity.

These patterns of “isotemporal displacement” have received very little attention in the physical activity literature. Very little is known about how people’s global use of time changes before, during and after an exercise intervention. The aim of this study was to investigate how previously inactive adults who had participated in a structured, partly supervised 6-week exercise program restructured their time budgets when the program ended. This study was conducted within a larger randomised controlled trial aimed at investigating the activitystat hypothesis [10].

## Methods

This study used a randomised controlled trial, multi-arm, parallel design, with two intervention groups and one control group and ethics approval was gained from the University of South Australia Human Research Ethics Committee (P009/10). Participants were allocated to groups on a 1:1:1 ratio using a computer generated random allocation sequence by a person external to the study. This study was registered with the Australian New Zealand Clinical Trials Registry (ACTRN12610000248066) and was conducted and is reported according to the CONSORT statement for reporting of randomised controlled trials [11]. Data collection was carried out at the University of South Australia, City East Campus, Adelaide, Australia. Participants were recruited in two cohorts, the first recruited in January 2011 with the final follow up in July/August 2011 and the second recruited in July 2011 with the final follow up in February 2012. No deviations from the study protocol relevant to this manuscript were made after the trial was commenced and the study protocol and CONSORT checklist for this paper are included as supporting information; see S1 Protocol and S1 Checklist.

### Participants

**Fig 1:**
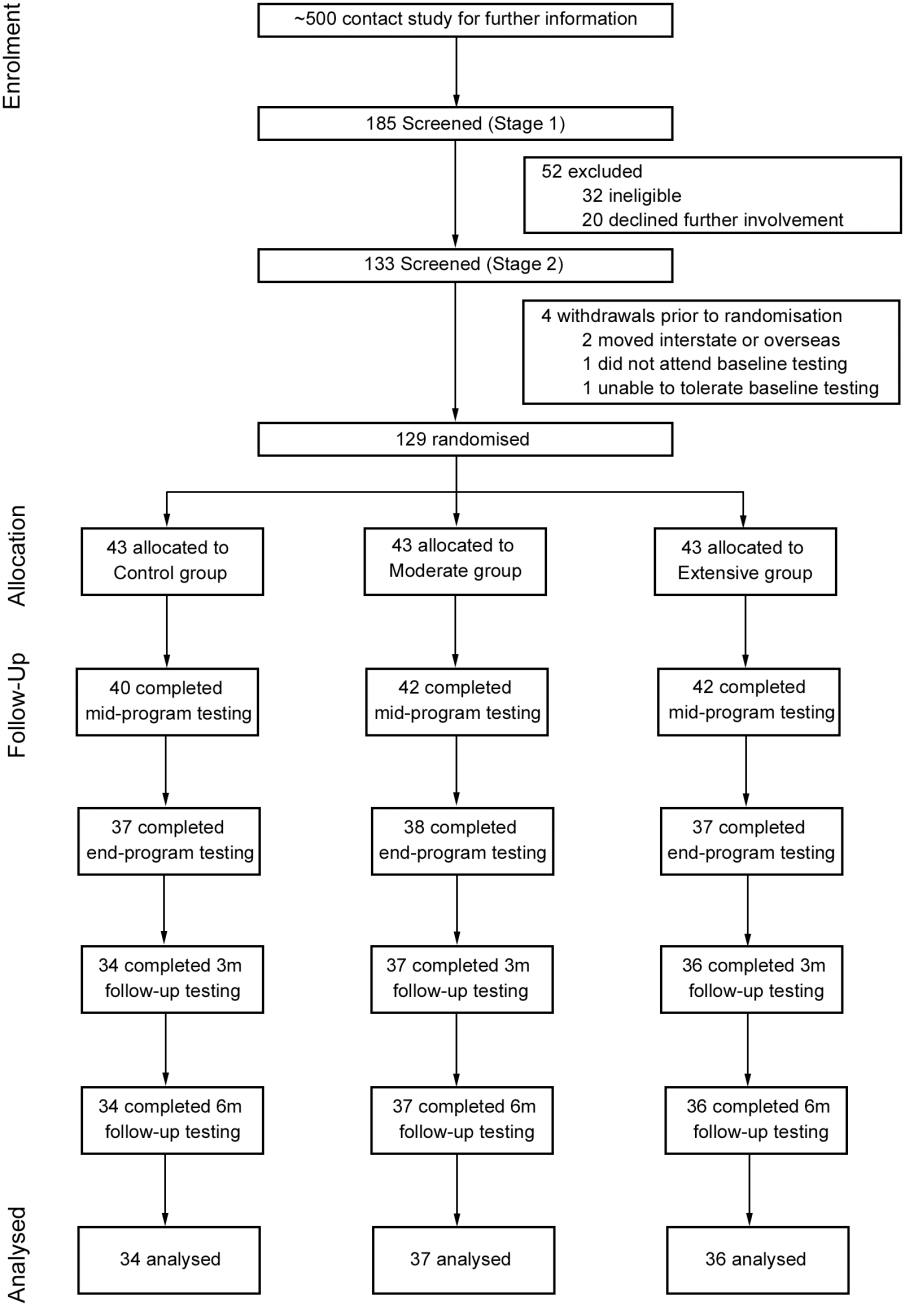
CONSORT flow diagram of participant recruitment, enrolment and progression through the study. *Note*: 3m = 3-month, 6m = 6-month.

Volunteers were recruited via email and print advertising from a large metropolitan university and several government departments. Prior to screening or testing, potential participants were required to provide written informed consent. For inclusion in this study, participants were required to be aged 18–60 years, weigh less than 150 kg, be insufficiently active [accumulating less than 150 minutes of moderate to vigorous physical activity (MVPA) in the past week according to the Active Australia Survey [12] (screening stage 1, Fig 1) and medically cleared for exercise under the Sports Medicine Australia pre-exercise screening criteria [13] (screening stage 2, Fig 1). Participants were provided with a $200 gratuity at completion of the study.

### The intervention

Participants in the two intervention groups (Moderate and Extensive) took part in an existing 40-day (6-week) physical activity program [14] located at the City East Campus of the University of South Australia. The goal of the physical activity program was to increase moderate to vigorous physical activity by 150 min/week in the Moderate group and 300 min/week in the Extensive group. The two intervention conditions involved similar types of activities and intensities and differed only in volume. Full details of the intervention have been provided elsewhere [10]. Half of the prescribed physical activity was to be accumulated in structured, supervised group classes, and half in the participants’ own time using modalities of their choice. The program was of moderate to vigorous intensity (60–85% HR_max_) and intensity was graded over the course of the 6-week program. The group sessions were designed to expend approximately 800 kJ per session in the first week and to increase by about 200 kJ in each subsequent week. The supervised sessions consisted of a wide variety of group activities such as circuit classes, boxing, dancing, bushwalking and kayaking. All sessions, both group and individual, were monitored for compliance using heart rate monitors (Polar S6101i, Finland). Participants allocated to the Control group were not given any instructions about what they should do. Participants in the Moderate and Extensive groups were given no specific instructions at the end of the 6-week physical activity intervention.

### Measurements

All participants undertook testing on five occasions: baseline (week 0), mid-program (weeks 3–4), end-program (week 6), and at 3- and 6-month follow-up (weeks 12 and 24). All outcome measures were administered by trained research assistants who were blinded to the participants’ group allocation. Measurements within the larger trial included use of time recalls, accelerometry, doubly labeled water, resting metabolic rate, VO_2max_, blood glucose and cholesterol, and anthropometry. The focus of this paper will be the use of time recalls and accelerometry data from all five measurement occasions.

#### Multimedia Activity Recall for Children and Adults

The adult version of the Multimedia Activity Recall for Children and Adults (MARCA) was used to capture use of time profiles of participants [15, 16]. The MARCA is a self-report, computerised 24-hour use-of-time recall tool that asks participants to recall everything they did in the last 24 hours from midnight to midnight, using meals as anchor points. Participants can choose from over 500 discrete activities, with the minimum time for an individual activity being five minutes. Each activity in the MARCA is assigned a metabolic equivalent (MET) value based on an expanded version of the Ainsworth compendium [17, 18], such that energy expenditure can be estimated. The adult version of the MARCA has test-retest reliabilities in adults of 0.920–0.997 for major activity sets such as sleep, physical activity and screen time, and convergent validity between physical activity level (PAL, estimated average rate of energy expenditure) and accelerometer counts/minute of rho = 0.72 [16]. A recent comparison of the children’s version with doubly-labeled water showed correlations of rho = 0.70 for total daily energy expenditure [19].

At each time point, the MARCA was administered by telephone by trained interviewers. Each time, two separate calls were made approximately one week apart, where in each call participants recalled the two previous days. This therefore resulted in four recalled days per measurement time point, where at least one weekday and one weekend day were required. Where possible, the same days of the week were recalled at all five time points.

#### Accelerometry

In addition to use of time recalls, accelerometry was used to objectively measure physical activity using the Actigraph GT3X accelerometer (Actigraph, Pensacola, Florida). The Actigraph GT3X has demonstrated very good intra- and inter-device reliability when tested using a motorised vibration table (intra-instrument coefficient of variation ≤2.5% and intra-class correlation coefficient of 0.97) [20, 21] and previous versions of the Actigraph (CSA and GT1M) have demonstrated hip-worn validity in treadmill walking and running compared with indirect calorimetry (r = 0.56, p<0.001 and r = 0.53, p<0.05, respectively) [22, 23]. Recent studies comparing the GT3X and GT1M accelerometer models have found similar results, with a concordance correlation coefficient between accelerometers of 0.99 [24]. At each of the five time points, participants were asked to wear the device on their right hip for 24 h per day, for seven days. Participants were advised to only remove the accelerometer overnight if they were unable to tolerate it while sleeping and for water-based activities. They were also asked to complete a brief log to record when and why they removed the monitor during the monitoring period. Accelerometers were programmed to capture 30-second epochs and minimum wear time was defined as 10 hours per day for four of the seven days, of which one must be a weekend day. Non-wear time was defined as 60 minutes of consecutive zeros.

### Data treatment

#### Multimedia Activity Recall for Children and Adults

Daily minutes of activity were calculated by summing the number of minutes participants reported being involved in each activity, and averaging them across the four recall days. The 520 activities in the MARCA were combined into “activity sets” and collapsed hierarchically into domains based on similarity and to preserve comparability with previous studies. Eleven mutually exclusive and exhaustive activity “superdomains” were identified: Physical Activity, Computer, Active Transport, Passive Transport, Quiet Time (e.g. reading and listening to music), Self-Care, Socio-cultural, Work and Study, Chores, Sleep, and TV/Videogames (previously published in greater detail [1]).

Activities were also clustered according to five mutually exclusive and exhaustive energy expenditure zones: 0–0.9 METs (sleep); 1–1.9 METs (very light physical activity, VLPA); 2–2.9 METs (light physical activity, LPA); 3–5.9 METs (moderate physical activity, MPA); and ≥6 METs (vigorous physical activity, VPA).

#### Accelerometry

Minutes spent in sleep/sedentary, light, moderate and vigorous physical activity domains were calculated using the Actilife 5.5 software (Actigraph, Pensacola, Florida), with any 30-second period above the count threshold included. Sleep and sedentary behaviour were combined due to the 24-h wear protocol used in this study. Minutes spent in the energy expenditure zones were calculated according to the vertical axis using cut points previously described by Troiano and colleagues and used in the United States National Health and Nutrition Examination Survey [25].

### Statistical analysis

Statistical analyses were conducted using SPSS version 21 (IBM Corporation, United States). Participants’ demographic characteristics were analysed descriptively but not formally tested for differences at baseline in accordance with the CONSORT guidelines for randomised controlled trials [11]. Differences in characteristics between completers and non-completers were analysed using Student’s t-test for continuous variables (age, body mass index and gross household income) and chi-squared tests for categorical variables (% female and group allocation). Compliance data (duration and intensity of physical activity sessions) based on objective heart rate monitoring during the intervention were analysed descriptively.

Because this study addresses mechanisms, analysis for the main outcomes was performed on a per protocol basis. Random effects mixed modeling (using the ‘Mixed Models/Generalised Linear Models’ function and a variance components covariance structure) was used to compare the variables of interest at each time point with time (0, 3, 6, 12 and 24 weeks) and group allocation (Control vs Moderate vs Extensive) as the fixed factors. For sub-group analyses only intervention participants were included with compliance [low complier (attended <70% of prescribed physical activity program) vs high complier (attended ≥70% of prescribed physical activity program)] as the fixed factor. Overall group and time p-values are reported, in addition to group x time interaction p-values for each time point. When there was a significant group x time interaction effect at a given time point, post-hoc analyses with Fisher’s least significant difference tests were used to identify where the significant effect was (e.g. Control vs Moderate group, Control vs Extensive group, Moderate vs Extensive group). Post-hoc findings are indicated by superscripts in the results tables. Where the data were skewed, generalised linear mixed models were applied according to the distribution. A significant group by time interaction indicated a significant difference in time use among the groups. Alpha was set at 0.05. While no correction has been made for multiple comparisons, actual p-values are reported.

A priori power calculations determined that with three groups and five measurement periods, random effects mixed modeling is able to detect a small effect size (Cohen’s d≥0.3) with 80% power and an alpha of 0.05 (two-sided) with 36 participants per group.

## Results

### Participant characteristics

A total of 129 participants were enrolled in the study, with 107 participants completing the study. The characteristics of the participants are shown in Table 1. A flow diagram of participant recruitment, enrolment and progression through the study is shown in Fig 1. Reasons for withdrawal included being unable to commit the time required to complete the study (n = 11), personal, work or family reasons (n = 7) or medical reasons (unrelated to the physical activity intervention; n = 4). Completers were more likely to be older (p<0.01; mean age of 43 years compared to 33 years for the non-completers). There was no statistical difference between completers and non-completers for gender (p = 0.22), gross household income (p = 0.88), body mass index (p = 0.74) or group allocation (Control, Moderate, Extensive; p = 0.68). Most were in full employment in mainly professional or clerical positions, 64% were women, and they came from households that were economically advantaged relative to the general Australian population.

**Table 1:** Baseline sociodemographic and anthropometric characteristics of the sample.

	Control	Moderate	Extensive
**N**	34	37	36
**Age (years)**	43 (10)	41 (12)	45 (10)
**Body Mass Index (kg/m** ^**2**^ **)**	26.1 (5.8)	25.4 (4.9)	26.8 (3.9)
**Household income** ^a^	104 (52)	106 (32)	97 (42)
**% Female**	59	64	67

^a^ Pre-tax income in thousands of Australian dollars per annum. One Australian dollar is approximately equivalent to one US dollar. The mean household income in Australia is about $70,000 p.a.

### Compliance with the intervention

Compliance with the prescribed physical activity program was measured by the duration and intensity of sessions recorded by objective heart rate monitoring. In accordance with the per protocol analysis, the following compliance data are presented for completers only (n = 36 Extensive group; n = 37 Moderate group). Duration was determined by weekly volume of physical activity sessions recorded using the heart rate monitor. Overall, participants in the Extensive group recorded on average 386 minutes per week and the Moderate group 195 minutes per week of physical activity. Intensity was determined on the basis of average heart rate for the entire recorded session (as a percentage of age-predicted maximal heart rate) for each participant. This included time spent in warm up and stretching activities and the cool-down period. Average weekly intensity in both groups ranged from approximately 65–75% HR_max_

### Changes following the physical activity program in superdomains of time use

**Fig 2:**
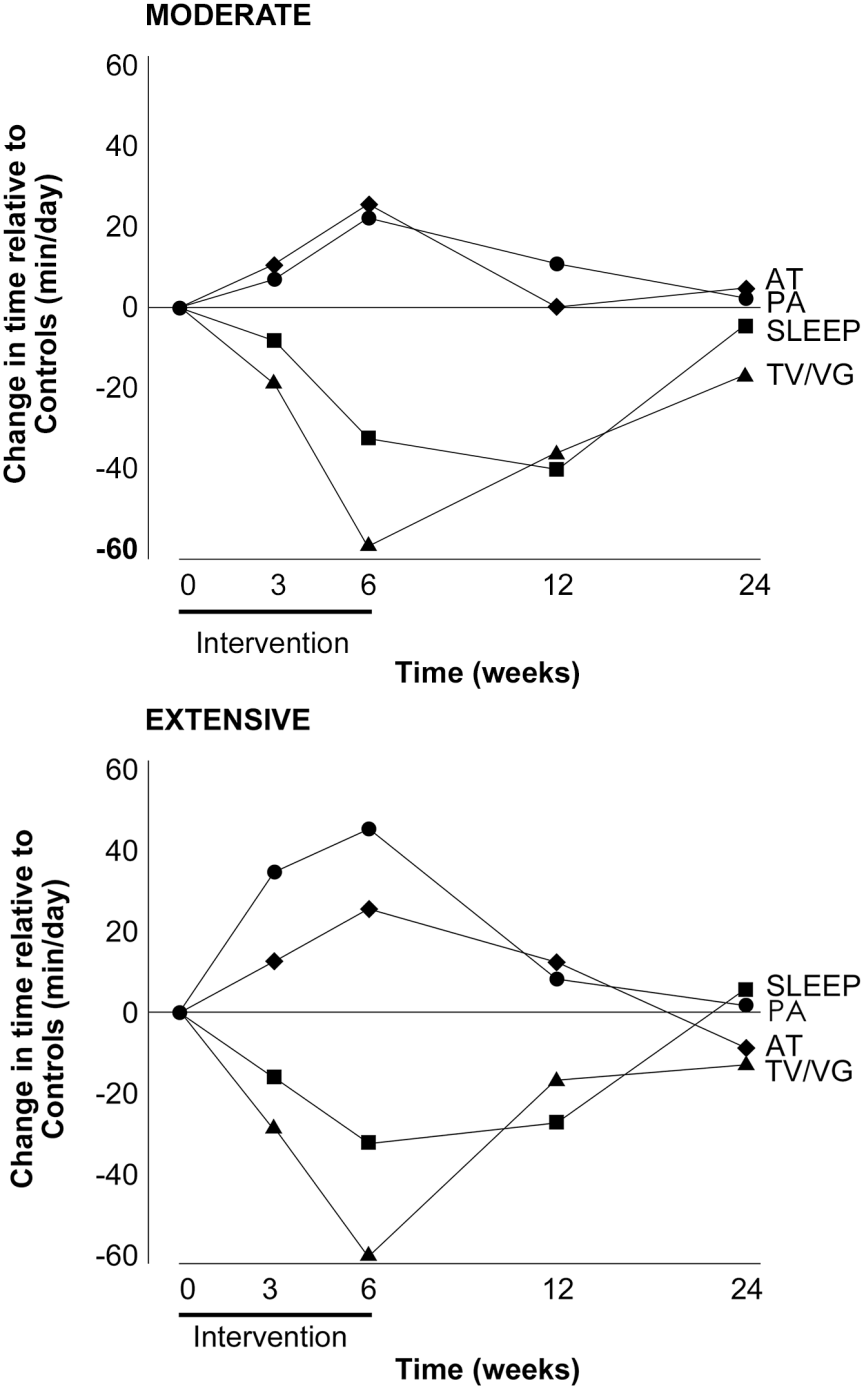
Changes in time from baseline to 6-month follow in the Moderate (top panel) and Extensive (bottom panel) groups, relative to Controls. *Note*: AT = Active Transport, PA = Physical Activity, TV/VG = Television/Videogames.

According to the MARCA, participants’ time use patterns had mostly returned to their pre-intervention pattern by 6-month follow up. The average number of minutes per day in each superdomain for each group at each of the five measurement occasions is shown in Table 2. The magnitude of the shifts in time among superdomains in the Moderate and Extensive groups, relative to Controls is shown in Table 3 and Fig 2.

**Table 2:** Mean (SD) time (min/d) spent in each superdomain by each of the groups at each time point, and P-values for main effects of Group and Time, and Group x Time interactions.

Super-domain	Period	Control	Moderate	Extensive	P	P	P
					Group	Time	Group x Time
**Physical Activity**	**Baseline**	6 (17)	10 (20)	10 (22)	**<0.001**	**<0.001**	0.06
	**Mid**	5 (14)^a^	15 (14)^a^	44 (25)^a^			**<0.001**
	**End**	2 (5)^ab^	27 (32)^a^	52 (42)^b^			**<0.001**
	**3 month**	3 (7)^ab^	18 (29)^a^	16 (28)^b^			**<0.001**
	**6 month**	7 (13)	13 (20)	12 (25)			0.49
**Computer**	**Baseline**	203 (131)	188 (113)	181 (105)	0.97	**0.002**	0.76
	**Mid**	153 (93)	146 (118)	166 (129)			0.82
	**End**	123 (88)	145 (109)	140 (95)			0.93
	**3 month**	173 (107)	182 (135)	178 (152)			0.95
	**6 month**	159 (114)	183 (118)	172 (121)			0.68
**Active Transport**	**Baseline**	52 (28)	57 (30)	59 (45)	**0.003**	0.13	0.65
	**Mid**	49 (32)^a^	63 (36)	70 (37)^a^			**0.02**
	**End**	39 (27)^ab^	67 (32)^a^	69 (30)^b^			**<0.001**
	**3 month**	51 (29)^a^	56 (28)	72 (49)^a^			0.08
	**6 month**	50 (28)	58 (36)	48 (27)			0.32
**Passive Transport**	**Baseline**	93 (36)^a^	72 (34)^a^	85 (44)	0.20	0.85	0.08
	**Mid**	85 (33)	81 (42)	83 (36)			0.82
	**End**	88 (43)	86 (40)	88 (43)			0.99
	**3 month**	100 (55)	80 (42)	83 (43)			0.17
	**6 month**	92 (34)	79 (51)	86 (44)			0.18
**Quiet Time**	**Baseline**	72 (50)^a^	65 (49)	45 (35)^a^	0.30	0.07	0.06
	**Mid**	59 (50)	47 (41)	48 (38)			0.26
	**End**	63 (70)	62 (51)	48 (53)			0.42
	**3 month**	54 (55)	55 (54)	47 (34)			0.78
	**6 month**	67 (48)	57 (49)	56 (47)			0.47
**Self-care**	**Baseline**	124 (24)	115 (38)	119 (28)	0.38	0.38	0.47
	**Mid**	122 (31)	122 (41)	126 (30)			0.86
	**End**	121 (33)	117 (31)^a^	135 (37)^a^			**0.04**
	**3 month**	122 (26)	125 (38)	131 (36)			0.56
	**6 month**	127 (24)	118 (37)	124 (31)			0.57
**Socio-cultural**	**Baseline**	106 (77)	119 (79)	113 (72)	0.52	0.06	0.71
	**Mid**	100 (64)	116 (81)	108 (73)			0.86
	**End**	96 (75)	110 (62)	99 (61)			0.48
	**3 month**	92 (63)	103 (90)	94 (68)			0.77
	**6 month**	103 (75)	120 (75)	114 (76)			0.72
**Work and Study**	**Baseline**	72 (77)	69 (79)	78 (78)	0.99	0.19	0.86
	**Mid**	113 (103)	95 (97)	92 (101)			0.77
	**End**	103 (104)	95 (106)	89 (78)			0.77
	**3 month**	79 (97)	109 (119)	78 (95)			0.24
	**6 month**	99 (102)	80 (88)	76 (68)			0.84
**Chores**	**Baseline**	133 (65)	118 (76)	138 (96)	0.18	0.83	0.47
	**Mid**	149 (72)^a^	124 (51)	111 (86)^a^			**0.05**
	**End**	155 (75)	118 (65)	131 (89)			0.18
	**3 month**	144 (69)	116 (63)	128 (97)			0.32
	**6 month**	150 (84)	119 (65)	131 (83)			0.44
**Sleep**	**Baseline**	467 (36)^a^	505 (84)^a^	483 (66)	0.19	0.10	0.09
	**Mid**	493 (52)	526 (72)^a^	492 (59)^a^			0.08
	**End**	508 (51)	514 (85)	492 (63)			0.44
	**3 month**	501 (71)	500 (76)	492 (77)			0.85
	**6 month**	474 (55)^a^	508 (61)^a^	506 (125)			0.09
**TV/Video games**	**Baseline**	112 (59)	122 (70)	129 (88)	0.59	0.23	0.97
	**Mid**	112 (70)	104 (61)	101 (74)			0.77
	**End**	144 (90)^ab^	98 (52)^a^	98 (80)^b^			**0.04**
	**3 month**	120 (64)	97 (61)	120 (78)			0.33
	**6 month**	112 (78)	105 (58)	116 (78)			0.88

Note: Summary data are raw scores and significant differences are indicated in **bold**. N = 107 (Control group, n = 34, Moderate group, n = 37, Extensive group, n = 36). Values with the same superscript were significantly different on post-hoc analysis. TV = television.

**Table 3:** Changes in time (min/d) spent in the 11 superdomains in the Moderate and Extensive groups relative to the Control group at mid-program, end-program, and 3 and 6 months follow-up.

	Change in use of time superdomains relative to Control:							
Superdomain	Moderate				Extensive			
	*Mid*	*End*	*3M*	*6M*	*Mid*	*End*	*3M*	*6M*
**Physical Activity**	**+6**	**+21**	**+11**	+2	**+35**	**+46**	**+9**	+1
**Computer**	+8	+37	+24	+39	+35	+39	+27	+35
**Active Transport**	+9	**+23**	0	+3	**+14**	**+23**	+14	–9
**Passive Transport**	+17	+19	+1	+8	+6	+8	–9	+2
**Quiet Time**	–5	+6	+8	–3	+16	+12	+20	+16
**Self-care**	+9	**+5**	+12	0	+9	**+19**	+14	+2
**Socio-cultural**	+3	+1	–2	+4	+1	–4	–5	+4
**Work & Study**	–15	–5	+33	–16	–27	–20	–7	–29
**Chores**	–10	–22	–13	–16	**–43**	–29	–21	–24
**Sleep**	–5	–32	–39	–4	–17	–32	–25	+6
**TV/Videogames**	–19	**–57**	–34	–18	–28	**–63**	–17	–13

Note: 3M = 3 months, 6M = 6 months. Significant differences according to Table 2 are shown in bold.

At the end of the intervention, the Moderate and Extensive groups were spending significantly more time in several time use domains including Physical Activity (21–46 min/d), Self-Care (5–19 min/d) and Active Transport (23 min/d in both groups) (Table 3). These changes were compensated by significant reductions in Television/Videogames (57–63 min/d). At the final follow up, all changes had reversed to within 10–15 min/d of baseline, with no significant changes retained at six months. Large but non-significant shifts in time were also seen in an increase in Computer time (24–27 min/d) and a decrease in Sleep (25–39 min/d) in the intervention groups, compared to the Control groups at the end of the intervention. By six month follow up, Sleep had returned to within ±6 min/d however Computer use remained elevated (39–45 min/d), albeit non-significantly.

### Changes following the physical activity program in different energy expenditure zones

**Fig 3:**
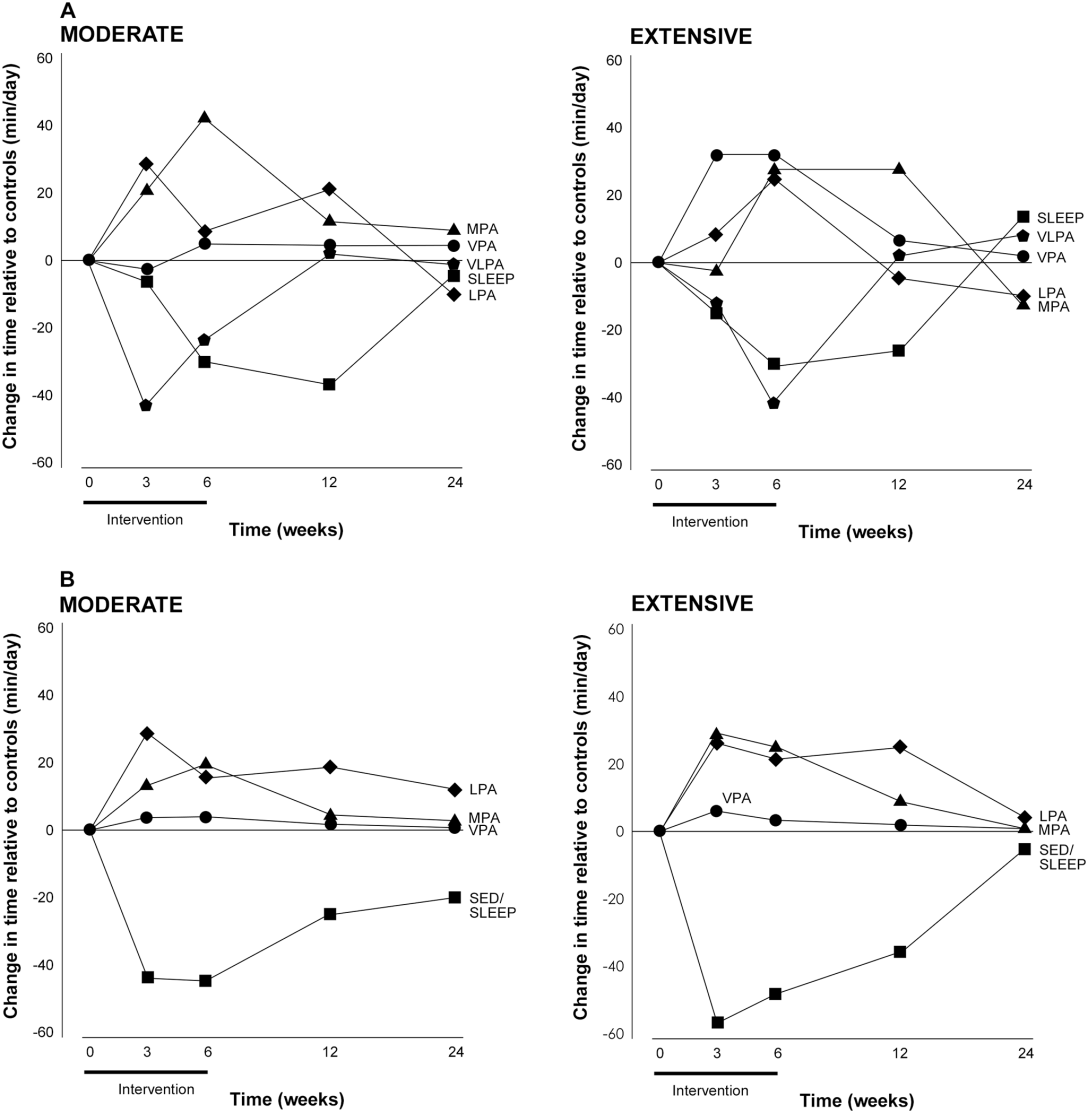
Changes in time (min/d) relative to Controls, from baseline to 6 months follow-up in time spent by the Moderate (left panel) and Extensive (right panel) groups in the different energy expenditure zones according to MARCA recalls (panel A) and accelerometry (Panel B). *Note*: SED/SLEEP = sedentary/sleep, VLPA = very light physical activity, LPA = light physical activity, MPA = moderate physical activity, VPA = vigorous physical activity.

Time spent in different energy expenditure zones was measured by the MARCA and accelerometry and results from both measures showed similar trends. The mean daily time spent within each energy expenditure zone, by each group at each time period according to the MARCA and accelerometry is shown in Table 4. The magnitude of the shifts in time among energy expenditure zones in the Moderate and Extensive groups, relative to Controls is shown in Table 5. Fig 3 compares the shifts in time according to both measures.

**Table 4:** Mean (SD) time (min/d) spent in each energy expenditure zone by each of the groups at each time point according to MARCA and accelerometry, and P-values for main effects of Group and Time, and Group x Time interactions.

EE Zone	Period	Control	Moderate	Extensive	P	P	P
					Group	Time	Group x Time
**MARCA**							
**Sleep (<1.0 METs)**	**Baseline**	467 (36)	505 (84)	483 (66)	0.61	0.09	0.46
	**Mid**	493 (52)	526 (72)	492 (59)			0.35
	**End**	508 (51)	514 (85)	492 (63)			0.24
	**3 month**	501 (71)	500 (76)	492 (77)			0.74
	**6 month**	474 (55)	508 (61)	506 (125)			0.28
**VLPA (1.0–1.9 METs)**	**Baseline**	662 (75)	655 (85)	641 (108)	0.37	**<0.0001**	0.56
	**Mid**	634 (96)	584 (98)	598 (120)			0.34
	**End**	630 (112)^a^	598 (109)	568 (109)^a^			0.08
	**3 month**	627 (106)	621 (92)	607 (131)			0.71
	**6 month**	638 (104)	630 (85)	626 (120)			0.93
**LPA (2.0–2.9 METs)**	**Baseline**	200 (74)	172 (60)	196 (68)	0.48	0.29	0.36
	**Mid**	201 (64)	202 (64)	203 (80)			0.98
	**End**	204 (70)	185 (61)^a^	222 (82)^a^			0.13
	**3 month**	203 (77)	198 (64)	193 (72)			0.86
	**6 month**	211 (77)^a^	173 (67)^a^	196 (65)			0.10
**MPA (3.0–5.9 METs)**	**Baseline**	108 (47)	99 (44)	114 (65)	0.50	0.59	0.47
	**Mid**	104 (53)	118 (77)	109 (39)			0.97
	**End**	88 (45)^ab^	121 (60)^a^	117 (53)^b^			**0.02**
	**3 month**	102 (49)	106 (70)^a^	131 (84)^a^			0.09
	**6 month**	108 (55)	109 (56)	100 (53)			0.78
**VPA (≥6.0 METs)**	**Baseline**	3 (8)^a^	9 (19)^a^	6 (11)	**<0.0001**	**<0.0001**	**0.01**
	**Mid**	8 (14)^a^	11 (12)^b^	38 (25)^ab^			**0.003**
	**End**	11 (29)^a^	21 (36)	41 (21)^a^			**0.001**
	**3 month**	7 (20)^a^	16 (33)	16 (26)^a^			**0.01**
	**6 month**	8 (13)	20 (51)	12 (18)			0.37
**ACCELEROMETRY**							
**Sedentary / Sleep (≤100 counts/min)**	**Baseline**	1,155 (76)	1,183 (64)	1,162 (70)	0.19	**<0.001**	0.22
	**Mid**	1,168 (61)^a^	1,154 (67)^b^	1,120 (74)^ab^			**0.02**
	**End**	1,163 (58)^a^	1,148 (60)	1,122 (63)^a^			**0.04**
	**3 month**	1,178 (62)	1,181 (65)	1,147 (72)			0.12
	**6 month**	1,181 (54)	1,189 (65)	1,182 (80)			0.87
**LPA (101–2019 counts/min)**	**Baseline**	250 (68)	223 (58)	243 (68)	0.40	**<0.001**	0.17
	**Mid**	234 (57)	233 (58)	250 (67)			0.46
	**End**	245 (53)	235 (52)	259 (57)			0.30
	**3 month**	230 (58)	222 (54)	248 (70)			0.27
	**6 month**	225 (49)	212 (58)	221 (75)			0.68
**MPA (2020–5998 counts/min)**	**Baseline**	34 (16)	33 (13)	35 (10)	**<0.001**	**<0.001**	0.92
	**Mid**	34 (15)^a^	44 (15)^a^	59 (15)^a^			**<0.001**
	**End**	32 (14)^ab^	51 (16)^a^	56 (23)^b^			**<0.001**
	**3 month**	32 (13)^a^	35 (17)	41 (16)^a^			0.08
	**6 month**	33 (18)	35 (15)	35 (16)			0.84
**VPA (≥5999 counts/min)**	**Baseline**	0.8 (2.1)	0.8 (1.9)	0.7 (1.7)	**<0.001**	**<0.001**	0.84
	**Mid**	0.7 (1.0)^ab^	4.0 (5.4)^a^	5.1 (6.6)^b^			**<0.001**
	**End**	0.5 (0.9)^ab^	3.9 (5.4)^a^	3.3 (4.9)^b^			**<0.001**
	**3 month**	0.4 (0.7)^ab^	1.5 (2.1)^a^	1.9 (3.0)^b^			**<0.001**
	**6 month**	0.7 (2.5)^ab^	2.0 (4.7)^a^	1.5 (4.0)^b^			**0.01**

Note: Summary data are raw scores and significant differences are indicated in **bold**. MARCA; N = 107 (Control group, n = 34, Moderate group, n = 37, Extensive group, n = 36). Accelerometry; N = 95 (Control group, n = 28, Moderate group, n = 35, Extensive group, n = 32). Average number of monitored days for included accelerometry data are baseline −6.9±0.5 days, mid −6.8± 0.8 days, end −6.6±1.5 days, 3 month −6.6±1.4 days, 6 month −6.7±1.1 days. Values with the same superscript were significantly different on post-hoc analysis. EE = energy expenditure, METs = metabolic equivalents, VLPA = very light physical activity, LPA = light physical activity, MPA = moderate physical activity, VPA = vigorous physical activity.

**Table 5:** Changes in time (min/d) spent in energy expenditure zones in the Moderate and Extensive groups relative to the Control group at mid-program, end-program and 3- and 6-month follow up according to the MARCA and accelerometry.

	Change in times spent in EE zones relative to Control:							
EE Zone	Moderate				Extensive			
	*Mid*	*End*	*3M*	*6M*	*Mid*	*End*	*3M*	*6M*
**MARCA**								
**Sleep (0.9 METs)**	–5	–32	–39	–4	–17	–32	–25	+16
**VLPA (1–1.9 METs)**	–43	–25	+1	-1	–15	–41	+1	+9
**LPA (2–2.9 METs)**	+29	+9	+23	–10	+6	+22	–6	–11
**MPA (3–5.9 METs)**	+23	**+42**	+13	+10	-1	**+23**	+23	–14
**VPA (≥6 METs)**	**–3**	+4	+3	+6	**+27**	**+27**	**+6**	+1
**ACCELEROMETRY**								
**Sedentary/Sleep (≤100 counts/min)**	**-42**	-43	-25	-20	**-55**	**-48**	-38	-6
**LPA (101–2019 counts/min)**	+26	+17	+19	+14	+23	+21	+25	+3
**MPA (2020–5998 counts/min)**	**+11**	**+20**	+4	+3	**+24**	**+23**	+8	+1
**VPA (≥5999 counts/min)**	**+3**	**+3**	**+1**	**+1**	**+5**	**+3**	**+2**	**+1**

Note: 3M = 3 months, 6M = 6 months. Significant differences according to Table 4 are shown in bold. EE = energy expenditure, METs = metabolic equivalents, VLPA = very light physical activity, LPA = light physical activity, MPA = moderate physical activity, VPA = vigorous physical activity.

During the intervention, time spent in moderate and vigorous energy expenditure zones increased, reaching significance in both accelerometry and MARCA estimates. To compensate for the increased time in moderate and vigorous energy expenditure zones, time spent in the sedentary zone decreased, reaching significance according to accelerometry estimates. At six month follow up, changes in time in sedentary and moderate zones were no longer significant, although increases in time in the vigorous zone remained significantly elevated by 0.86–1.25 min/d in the intervention groups compared to the Control group, according to accelerometry.

## Discussion

### Key findings

The aim of this study was to investigate how previously inactive adults who had participated in a structured, partly supervised 6-week physical activity program restructured their time budgets when the program ended. This was investigated in two ways, firstly by exploring how time in specific activity domains was restructured and secondly, how time spent in mutually exclusive energy expenditure zones was restructured. Changes during the intervention are examined in more detail in our previous work [1].

At the end of the intervention, relative to baseline and Controls, participants in the Moderate and Extensive group were participating in an additional 147 and 322 minutes of physical activity per week according to MARCA estimates. This is largely commensurate with the prescribed physical activity program of an additional 150 and 300 minutes per week. The main finding of this study was that following this imposed physical activity program, both time spent in major domains of time use and time spent in energy expenditure zones largely returned to pre-program levels within 20 weeks after the end of the program. All significant changes found during the intervention were no longer significant at 6-month follow up, with the exception of time spent in vigorous physical activity (when measured by accelerometry), which remained statistically elevated, albeit small in magnitude (0.9–1.3 min/d), in both intervention groups, compared to controls. The results of this study demonstrate a strong pattern of recidivism and confirm previous research that maintaining physical activity participation following a structured, group-based physical activity program is challenging [2, 26].

### Implications

#### Possible explanations of time shifts

While recidivism is a well-known phenomenon among people undertaking physical activity programs, this study shows similar “recidivist” patterns in aspects of time use other than physical activity. These results raise the possibility that physical activity recidivism in interventions could be driven by the activities that have been displaced. Time use literature commonly divides time into obligatory time (time spent in paid and unpaid labour and personal care, such as sleeping, grooming and eating) and discretionary time (the time remaining after obligatory time has been accounted for) [27]. The results of the current study suggest that time for a new physical activity program is drawn from discretionary time, for example television viewing. It is surprising, however, to also identify a trend for a relative Sleep reduction in the intervention groups, as Sleep is an activity that is not classically considered as discretionary.

Large, albeit non-significant, shifts in Sleep were demonstrated in both the Moderate and Extensive groups, resulting in Sleep deficits of -39 and -32 min/d, relative to Controls. While these changes are largely driven by changes in the Control group (+46 min/d in Sleep at end intervention) with small absolute changes in Sleep in the intervention groups, this does not necessarily entail that there is not a sleep deficit, as sleep requirements can, and probably do, change across the year. This finding is also supported by subgroup analyses which revealed that intervention participants who complied with at least 70% of the physical activity program slept approximately 40 min/day less than non-compliers (p<0.01) (S1 Table).

The sustainability of decreasing time spent in obligatory activities is not well understood. Chronic reduction in sleep duration has been coined a ‘sleep debt’ that in the short term, can be tolerated, but in the longer term is accounted for by making up lost time [28]. These ‘time debts’ could also be incurred in other areas, such as television viewing. While typically considered discretionary time, individuals may need a certain amount of relaxation time, which they may accumulate through television viewing time [29]. There is a possibility that reversing the accumulated ‘time debt’, is what drives the return to baseline after an imposed stimulus and that addressing this drift back to baseline behaviours could be explored in future studies.

The specificity of the return to baseline levels, in most variables within 20 min/d, may also suggest a habitual preference for time use. One possible explanation is that the intervention was not a sufficient stimulus to overcome existing behavioural habits and to integrate habitual physical activity. Starting a new exercise program results in physical activity that is intentional and goal directed. However, individuals who engage in regular physical activity often exhibit habitual responses, where physical activity is triggered by precursory environmental stimuli rather than an expectant outcome [30]. Over time, this transition of action to habit is reflective of neurobiological plasticity [31]. Evidence suggests that the time for a behaviour to transition to habit is highly variable by individual, the complexity of the behaviour and the circumstances in which the behaviour is being adopted [32]. Perhaps an intervention with different parameters, such as longer length (>6 weeks) [32], would allow for the transition of changes in behaviour from being intentional to habitual and therefore resulting in long-term participation.

#### Implications for the design of interventions

While the current study demonstrated that physical activity can be increased in previously sedentary adults, it also confirms previous research that these are often temporary changes [2]. Translating these temporary changes into long-term physical activity participation continues to be a challenge for physical activity research. This study raises the possibility that activities that are displaced when trying to increase a target activity, such as physical activity, may influence the sustainability of behaviour change over time. Rather than conceptualising behaviour change as affecting only one activity, we should think in terms of patterns of change and what patterns may be more sustainable than others.

For example, in the current study, the significant increase in active transport was not associated with a subsequent decrease in passive transport in the intervention groups. Therefore, it is likely that active transport took the form of extra trips for the physical activity program. If active transport had instead displaced passive transport (e.g. commuting to work by bicycle), this may be more sustainable over time than simply adding in active transport for the duration of the intervention. To conceptualise and educate about patterns of change, it is important that in future physical activity interventions, all aspects of time use are measured, not just the activity of interest.

Additionally, while there was a downward trend in the time dedicated to active transport and physical activity at three months, changes in Physical Activity remained statistically elevated relative to Controls. Future physical activity intervention research may therefore consider small ‘booster shots’ or ‘prompts’ of physical activity at approximately three monthly intervals. Booster shots of activity may be more effective in nudging individuals’ baseline physical activity levels into ‘sufficiently active’ ranges where health benefits can be attained [33].

### Strengths and limitations

There are very few studies which comprehensively track time use changes across a physical activity intervention. This study used a validated, reliable, high-resolution 24-hour recall exhaustively covering all activity domains. Use of time was assessed before, during and after the physical activity program and a randomised controlled trial design was employed, allowing us to filter out changes in use of time due to seasonal, maturational and environmental effects [34].

Nevertheless, this study had a number of limitations. It used a sample of convenience. The study sample was predominantly female, well educated, generally in full employment and were relatively active at baseline. Completers were significantly older than non-completers and the results cannot necessarily be generalised to other groups with different time commitments and constraints. It is also important to emphasise that use of time among the Control group varied a great deal across the study period, and the difference reported here are relative to the Control group. Self-report data may also be subject to social desirability bias and recall errors, though the constraint of having to account for all 24 hours in the day minimises these risks. Furthermore, the strong agreement between MARCA-estimated changes in time use and changes calculated from accelerometry data (Fig 2) supports the validity of the self-report measure.

The participants all exceeded the recommended levels of MVPA at baseline (>150 min/wk) according to both MARCA and accelerometry estimates, therefore exceeding the inclusion criteria for this study, in spite of prior screening using the Active Australia instrument. There are three explanations for this, (1) the Active Australia questionnaire underestimates MVPA compared to other physical activity measures, (2) the participants could have changed their activity levels between screening and formal enrolment in the study and (3) that some participants intentionally underreported their MVPA during screening to be included in the study. We are unable to determine which explanation is most likely but this may have limited the initial compliance with the physical activity program, and limits the generalisability of the findings.

Finally, the current paper has examined changes in time use following an exercise program at a group mean level, however it is likely that individual patterns of behavior are variable and may reveal further insights into maintenance of physical activity behaviours. This is an area for future research and the findings from the current study suggest that changes in sleep and television viewing time are key domains of time use to explore in more detail [35].

## Conclusion

While the current study demonstrated that physical activity can be increased in previouslysedentary adults, it also confirms previous research that these are often temporary changes.

Translating these temporary changes into long-term physical activity participation continues to be a challenge for physical activity research. While a six-week imposed physical activity program generated shifts in time use and energy expenditure, time use patterns returned to baseline within 20 weeks of the end of the program. Examining how time budgets are rearranged following an physical activity intervention is a novel method and has highlighted that physical activity must be considered in the context of other aspects of time use, which may have their own rhythms and pressures. In designing and determining the effectiveness of physical interventions, it is therefore important to consider the whole profile of time use, rather than focusing on individual activities.

## Supporting Information

S1 Protocol Study protocol. (PDF)

S1 Checklist CONSORT checklist. (PDF)

S1 Table Time (min/day) spent in each time use superdomain in low compliers compared with high compliers, measured by the MARCA. Note: Summary data are raw scores and significant differences are indicated in bold. Sample includes intervention participants only sub-divided into low compliers (attended <70% of prescribed physical activity program) and high compliers (attended ≥70% of prescribed physical activity program). N = 73 (Low compliers, n = 37, High compliers n = 36). SD = standard deviation, TV = television.
(PDF)

S1 Dataset Manuscript data. (XLSX)

## References

[1] Gomersall S, Norton K, Maher C, English C, Olds T. In search of lost time: when people undertake a new exercise program, where does the time come from? A randomized controlled trial. J Sci Med Sport. 2015;18(1):43–8. 10.1016/j.jsams.2014.01.004 24602689

[2] Seefeldt V, Malina RM, Clark MA. Factors affecting levels of physical activity in adults. Sports Med. 2002;32(3):143–68. 11839079 10.2165/00007256-200232030-00001

[3] Liira H, Engberg E, Leppävuori J, From S, Kautiainen H, Liira J, et al Exercise intervention and health checks for middle-aged men with elevated cardiovascular risk: a randomized controlled trial. Scand J Prim Health Care. 2014:1–7. 10.3109/02813432.2014.984967 25434409 PMC4278388

[4] Conroy M, Sward K, Spadaro K, Tudorascu D, Karpov I, Jones B, et al Effectiveness of a physical activity and weight loss intervention for middle-aged women: healthy bodies, healthy hearts randomized trial. J Gen Intern Med. 2014:1–7. 10.1007/s11606-014-3077-5 25391601 PMC4314485

[5] Healy GN, Matthews CE, Dunstan DW, Winkler EAH, Owen N. Sedentary time and cardio-metabolic biomarkers in US adults: NHANES 2003–06. Eur Heart J. 2011;32(5):590–7. 10.1093/eurheartj/ehq451 21224291 PMC3634159

[6] Veerman JL, Healy GN, Cobiac LJ, Vos T, Winkler EAH, Owen N, et al Television viewing time and reduced life expectancy: a life table analysis. Br J Sports Med. 2011;46(13):927–30. 10.1136/bjsm.2011.085662 23007179

[7] Cappuccio FP, Taggart FM, Kandala NB, Currie A, Peile E, Stranges S, et al Meta-analysis of short sleep duration and obesity in children and adults. Sleep. 2008;31(5):619–26. 18517032 10.1093/sleep/31.5.619 PMC2398753

[8] Tsuno N, Besset A, Ritchie K. Sleep and depression. J Clin Psychiatry. 2005;66(10):1254–69. 16259539 10.4088/jcp.v66n1008

[9] Rowland TW. The biological basis of physical activity. Med Sci Sports Exerc. 1998;30(3):392–9. 9526885 10.1097/00005768-199803000-00009

[10] Gomersall S, Maher CN, K., Dollman J, Tomkinson G, Esterman A, English C, et al Testing the activitystat hypothesis: a randomised controlled trial protocol. BMC Public Health. 2012;12:851 10.1186/1471-2458-12-851 23043381 PMC3503831

[11] Moher D, Hopewell S, Schulz KF, Montori V, Gøtzsche PC, Devereaux PJ, et al CONSORT 2010 Explanation and Elaboration: updated guidelines for reporting parallel group randomised trials. Br Med J. 2010;340:c869 10.1136/bmj.c869 20332511 PMC2844943

[12] Australian Institute for Health and Welfare. The Active Australia survey: a guide and manual for implementation, analysis and reporting Canberra, Australia: Australian Institute for Health and Welfare; 2003 [14 January 2011]. Available: http://www.aihw.gov.au/publication-detail/?id=6442467449.

[13] Sports Medicine Australia. Sports Medicine Australia pre-exercise screening system: Sports Medicine Australia; 2009 [cited 2012 18 July]. Available from: http://www.sma.org.au/wpcontent/uploads/2009/05/new_pre_screening.pdf.

[14] Norton L, Norton K, Lewis N, Dollman J. A comparison of two short-term intensive physical activity interventions: methodological considerations. International Journal of Behavioral Nutrition and Physical Activity. 2011;8:133 10.1186/1479-5868-8-133 22136578 PMC3240814

[15] Ridley K, Olds TS, Hill A. The multimedia activity recall for children and adolescents (MARCA): development and evaluation. International Journal of Behavioral Nutrition and Physical Activity. 2006;3:10 10.1186/1479-5868-3-10 16725055 PMC1524806

[16] Gomersall SR, Olds TS, Ridley K. Development and evaluation of an adult use-of-time instrument with an energy expenditure focus. J Sci Med Sport. 2011;14(2):143–8. 10.1016/j.jsams.2010.08.006 20932797

[17] Ainsworth BE, Haskell WL, Whitt MC, Irwin ML, Swartz AM, Strath SJ, et al Compendium of physical activities: an update of activity codes and MET intensities Med Sci Sports Exerc. 2000;32(9):S498–S516. 10993420 10.1097/00005768-200009001-00009

[18] Ainsworth BE, Haskell WL, Herrmann SD, Meckes N, Bassett DRJ, Tudor-Locke C, et al Compendium of physical activities: a second update of codes and MET values. Med Sci Sports Exerc. 2011;43(8):1575–81. 10.1249/MSS.0b013e31821ece12 21681120

[19] Foley L, Maddison R, Rush E, Olds T, Ridley K, Jiang Y. Doubly labeled water validation of a computerized use of time recall in active young people. Metabolism. 2013;62(1):163–9. 10.1016/j.metabol.2012.07.021 22980224

[20] Santos-Lozano A, Marín PJ, Torres-Luque G, Ruiz JR, Lucía A, Garatachea N. Technical variablity of the GT3X accelerometer Med Eng Phys. 2012;34:787–90. 10.1016/j.medengphy.2012.02.005 22417978

[21] Brage S, Wedderkopp N, Franks PW, Andersen LB, Froberg K. Re-examination of validity and reliability of the CSA monitor in walking and runnin. Med Sci Sports Exerc. 2003;35(8):1447–54 12900703 10.1249/01.MSS.0000079078.62035.EC

[22] Swartz AM, Strath SJ, Bassett DR, O’Brien WL, King GA, Ainsworth BE. Estimation of energy expenditure using CSA accelerometers at hip and wrist sites. Med Sci Sports Exerc. 2000;39(9):S450–S6. 10.1097/00005768-200009001-00003 10993414

[23] Abel M, Hannon J, Sell K, Lillie T, Conlin G, Anderson D. Validation of the Kenz Lifecorder EX and ActiGraph GT1M accelerometers for walking and running in adults. Applied Physiology, Nutrition and Metabolism. 2008;33(6):1155–64. 10.1139/h08-103 19088773

[24] Vanhelst J, Mikulovic J, Biui-Xuan G, Dieu O, Blondeau T, Fardy P, et al Comparison of two Actigraph accelerometer generations in the assessment of physical activity in free living conditions. BMC Research Notes. 2012;25(5):187–90. 10.1186/1756-0500-5-187 22534207 PMC3477007

[25] Troiano R, Berrigan D, Dodd KW, Mâsse LC, Tilert T, McDowell M. Physical activity in the United States measured by accelerometer. Med Sci Sports Exerc. 2008;40(1):181–8. 18091006 10.1249/mss.0b013e31815a51b3

[26] Foster C, Hillsdon M, Thorogood M. Interventions for promoting physical activity (review). Cochrane Database of Systematic Reviews. 2005;1 Art No.: CD003180 10.1002/14651858.CD003180.pub2 15674903 PMC4164373

[27] Goodin RE, Rice J.M., Bittman M, Saunders P. The time-pressure illusion: Discretionary time versus free time. New South Wales, Australia: The Social Policy Research Centre, University of New South Wales, 2002.

[28] Van Dongen HPA, Rogers NL, Dinges DF. Sleep debt: Theoretical and empirical issues. Sleep and Biological Rhythms. 2003;1:5–13.

[29] Weaver JB III. Individual differences in television viewing. Personality and Individual Differences. 2003;35:1427–37.

[30] Aarts H, Paulussen T, Schaalma H. Physical exercise habit: on the conceptualisation and formation of habitual health behaviours. Health Educ Res. 1997;12(3):363–74. 10174219 10.1093/her/12.3.363

[31] Yin HH, Knowlton BJ. The role of the basal ganglia in habit formation. Nature Reviews Neuroscience. 2006;7:464–76. 16715055 10.1038/nrn1919

[32] Lally P, Van Jaarsveld CHM, Potts HWW, Wardle J. How are habits formed: modelling habit formation in the real world. European Journal of Social Psychology. 2010;40:998–1009.

[33] Fjeldsoe B, Neuhaus M, Winkler E, Eakin EG. Systematic review of maintenance of behaviour change following physical activity and dietary interventions. Health Psychol. 2011;30(1):99–109. 10.1037/a0021974 21299298

[34] Gomersall S, Rowlands A, English C, Maher C, Olds T. The ActivityStat hypothesis: the concept, the evidence, and the methodologies. Sports Med. 2012;43(2):135–49. 10.1007/s40279-012-0008-7 23329607

[35] Hekler EB, Buman MP, Poothakandiyil N, Rivera DE, Dzierzewski JM, Morgan AA, et al Exploring behavioral markers of long-term physical activity maintenance: a case study of system identification modeling within a behavioral intervention. Health Educ Behav. 2013;40(10). 10.1177/1090198113496787 PMC3806212 24084400

