# Supplementary material for: Time Regained: When People Stop a Physical Activity Program, How Does Their Time Use Change? A Randomised Controlled Trial
Source: PLoS One. 2015 May 29;10(5):e0126665. doi: 10.1371/journal.pone.0126665 (PMC4449013; doi:10.1371/journal.pone.0126665)
Supplement: S1 Protocol — (PDF) [file pone.0126665.s001.pdf]

BMC Public Health

STUDY PROTOCOL Open Access

Testing the activitystat hypothesis: a randomised controlled trial protocol

Sjaan Gomersall[1*], Carol Maher[1], Kevin Norton[2], Jim Dollman[2], Grant Tomkinson[1], Adrian Esterman[3], Coralie English[4], Nicole Lewis[2] and Tim Olds[1]

Abstract

Background: The activitystat hypothesis proposes that when physical activity or energy expenditure is increased or decreased in one domain, there will be a compensatory change in another domain to maintain an overall, stable level of physical activity or energy expenditure. To date, there has been no experimental study primarily designed to test the activitystat hypothesis in adults. The aim of this trial is to determine the effect of two different imposed exercise loads on total daily energy expenditure and physical activity levels.

Methods: This study will be a randomised, multi-arm, parallel controlled trial. Insufficiently active adults (as determined by the Active Australia survey) aged 18–60 years old will be recruited for this study (n=146). Participants must also satisfy the Sports Medicine Australia Pre-Exercise Screening System and must weigh less than 150 kg. Participants will be randomly assigned to one of three groups using a computer-generated allocation sequence. Participants in the *Moderate* exercise group will receive an additional 150 minutes of moderate to vigorous physical activity per week for six weeks, and those in the *Extensive* exercise group will receive an additional 300 minutes of moderate to vigorous physical activity per week for six weeks. Exercise targets will be accumulated through both group and individual exercise sessions monitored by heart rate telemetry. Control participants will not be given any instructions regarding lifestyle. The primary outcome measures are activity energy expenditure (doubly labeled water) and physical activity (accelerometry). Secondary measures will include resting metabolic rate via indirect calorimetry, use of time, maximal oxygen consumption and several anthropometric and physiological measures. Outcome measures will be conducted at baseline (zero weeks), mid- and end-intervention (three and six weeks) with three (12 weeks) and six month (24 week) follow-up. All assessors will be blinded to group allocation.

Discussion: This protocol has been specifically designed to test the activitystat hypothesis while taking into account the key conceptual and methodological considerations of testing a biologically regulated homeostatic feedback loop. Results of this study will be an important addition to the growing literature and debate concerning the possible existence of an activitystat.

Trial registration: Australian New Zealand Clinical Trials Registry ACTRN12610000248066

Keywords: Protocol, Randomised controlled trial, Physical activity, Activitystat

Background

There are many well-documented benefits to participating in regular physical activity, with national guidelines on physical activity levels being implemented in many developed countries for a number of years [1-3]. However, interventions to increase physical activity continue to have limited success [4] and physical inactivity continues to be a major and costly contributor to the burden of disease, demonstrating a dose–response relationship with a number of poor heath outcomes including obesity, cardiovascular disease and type II diabetes [5,6]. One explanation for the limited success in improving physical activity levels is known as the 'activitystat' hypothesis.

First described in 1998, the activitystat hypothesis suggests that when physical activity (or perhaps energy expenditure) is increased or decreased in one domain, there will be a compensatory change in another domain,

* Correspondence: sjaan.gomersall@mymail.unisa.edu.au
[1]Health and Use of Time (HUT) Group, Sansom Institute for Health Research, University of South Australia, Adelaide, South Australia, Australia
Full list of author information is available at the end of the article

BioMed Central

© 2012 Gomersall et al.; licensee BioMed Central Ltd. This is an Open Access article distributed under the terms of the Creative Commons Attribution License (http://creativecommons.org/licenses/by/2.0), which permits unrestricted use, distribution, and reproduction in any medium, provided the original work is properly cited.

in order to maintain an overall stable level of physical activity or energy expenditure [7]. It was proposed that this mechanism is biologically regulated, with an activitystat taking on the characteristics of a homeostatic feedback loop, whereby a setpoint of physical activity or energy expenditure is regulated by compensatory adjustments through as yet undetermined mechanisms. The idea of the activitystat has challenged conventional wisdom regarding physical activity and physical activity interventions. As such, its existence, or not, has vital consequences for physical activity and public health research. If an activitystat truly exists, it would suggest that conventional behavioural physical activity interventions are destined to fail.

The postulation of an activitystat was primarily founded on plausible biological mechanisms in both humans and animals [8-11] and a small number of experimental studies which failed to demonstrate increased overall physical activity or energy expenditure with imposed physical activity loads [7]. A recent systematic review by Gomersall and colleagues [12] examined the current state of the activitystat literature. To date, much of the evidence for an activitystat is based on observational data from children where retrospective or secondary analyses have been used to question the existence of an activitystat. These studies have shown mixed results. The review identified 15 papers experimentally investigating compensation in adults, with 60% of these studies finding some evidence of compensation. However, the studies varied dramatically in their methodological parameters, study designs and sample sizes, type and duration of intervention, outcome measures and assumptions of the regulated variable. Due to the lack of consistency in methodological approach and findings, the review found that evidence for the presence or not of an activitystat is currently equivocal. It concluded that in order to advance the field, purpose-designed experimental studies with specific methodological considerations are required.

As outlined by the review, a purpose-designed study investigating the activitystat hypothesis should be experimental in nature and include an imposed stimulus for compensation. The study should employ objective measures of both physical activity and energy expenditure (including measures of resting metabolic rate) to ensure that both variants of the regulated variable are monitored. Ideally, this would include the gold standard methodology for measuring total energy expenditure in humans, doubly labeled water [13]. Given that the time period over which a supposed activitystat operates is currently unclear, the study should employ a sufficiently long stimulus with regular measurement periods to help identify the time course of compensation. Furthermore, the stimulus should be of a sufficient strength to induce compensation. Ideally this would include multiple physical activity stimuli of different strengths to allow investigation of the threshold of the activitystat mechanism. Compliance with the stimulus should be monitored and a per-protocol analysis should be employed, given that the study's purpose is to test the mechanism of the activitystat and not the effectiveness of an intervention. Participants must also not be at the extremes of very active or very sedentary. Participants who are very active may demonstrate a "ceiling effect" with neither the time nor the drive to add further physical activity to their schedules. Very inactive participants may demonstrate a "floor effect" where they may have insufficient physical activity in other domains to trade off against the imposed physical activity program. Finally, a randomised controlled trial design should be employed to account for the possibility of dynamic setpoints (due, for example, to maturation [14] and season [15]). To date, there is no study that comprehensively covers this methodological framework.

Therefore, this paper presents a protocol specifically designed to test the activitystat hypothesis in adults, taking into account these key methodological considerations. The primary aim of this study is to determine the effect of two different imposed 6-week exercise loads in previously sedentary 18–60 year olds on total energy expenditure and physical activity. The secondary aims of this study are (a) to determine how participants adjust their daily activity patterns to accommodate the imposed exercise load, and (b) to investigate dose–response relationships between physical activity and anthropometric and physiological variables.

Methods/design

'Testing the ActivityStat Hypothesis' is a multi-arm, parallel and single-blinded randomised controlled intervention trial. Participants will be allocated to one of three groups, a control group or one of two intervention groups: a *Moderate* exercise group or an *Extensive* exercise group.

Ethical approval

This study has received ethical approval from the University of South Australia Human Research Ethics Committee prior to study commencement and is registered with the Australian New Zealand Clinical Trials Registry (Trial Registration: ACTRN12610000248066). All participants will be required to provide written informed consent prior to screening assessments.

Participants

The participants will be males and females aged 18–60 years who meet the following selection criteria:

- Insufficiently active, defined as participating in less than 150 minutes of moderate to vigorous physical activity (MVPA) in the last week as determined by the Active Australia Survey [16]

- Satisfy the pre-exercise screening guidelines for commencing exercise as determined by the Sports Medicine Australia Pre-Exercise Screening System [17]
- Body weight <150 kg

Participants must weigh less than 150 kg due to the increased risk of injury during a moderate or extensive exercise program. Any potential participant who is excluded on this basis will be referred to the University of South Australia Exercise Physiology Clinic.

Recruitment and activity screening

Participants for this study will be recruited via email and print advertising in a metropolitan university, a tertiary hospital and several government departments. Potential participants will be encouraged to contact the researchers for further information and will then be forwarded details regarding participant commitments and expectations. Participants who remain interested will then be invited to attend the Exercise Research Laboratory at the University of South Australia to undertake the Active Australia Survey [16], a past week physical activity questionnaire. Total physical activity time will be calculated by adding the time spent in walking and moderate activity plus twice the vigorous activity time (not including gardening and housework). Individuals who report < 150 min of weighted physical activity per week will be considered "insufficiently active" and will be eligible for the study. Those who achieved ≥150 min/wk in the preceding week will be excluded. Included participants will then undertake a formal laboratory orientation to the testing protocols and laboratory environment and a second laboratory visit will be scheduled for pre-exercise screening, and health and fitness assessments.

Pre-exercise screening

Pre-exercise screening will be carried out using the Sports Medicine Australia Pre-Exercise Screening System [17]. This system involves a series of heath-related questions and physiological tests to determine whether the participant is recommended for medical clearance before commencing an exercise program. The system involves two stages. Stage 1, via questionnaire, classifies individuals as high risk for exercise-related complications due to known, or signs and symptoms of, cardiovascular, respiratory, or pulmonary disease. High risk individuals are recommended to obtain medical clearance prior to commencing exercise testing or an exercise program. Stage 2, via questionnaire and physiological testing, classifies individuals as moderate or low risk based on their age and the number of cardiovascular disease risk factors possessed. Both moderate and low risk individuals can commence a vigorous intensity exercise program and undergo maximal exercise testing post-screening, however, moderate risk individuals are recommended to obtain medical clearance beforehand. Individuals who obtain medical clearance or do not require it will progress to baseline testing. Those who fail to obtain medical clearance will be excluded from the study.

Randomisation and blinding

Participants will be randomly allocated to one of the three groups (Moderate exercise group, Extensive exercise group or Control) by a person external to the study using computer-generated allocation sequence. This will be carried out once baseline testing is completed to ensure that allocation concealment is satisfied. It is not possible to blind participants to group allocation due to the nature of physical activity, however all participants will be blinded to the activitystat hypothesis. All research assistants carrying out physical and questionnaire measures will be blinded to the participants' group allocation.

Intervention

The two intervention conditions are based upon a six week group-based physical activity intervention - a program previously developed and tested within the University of South Australia, known as the 40-DAY Physical Activity Program [18]. The program comprises both group-based, instructor-led and self-directed individual exercise sessions with progressive increases in exercise intensity. The two intervention conditions involve similar types of physical activities and intensities, and differ only in volume. The group sessions are graded in intensity and are designed to expend approximately 800 kJ in the first week and to increase by about 200 kJ per session in each subsequent week.

This intervention was designed within the framework of several theoretical components important for long-term behavioural change, including an intra-personal focus on self-efficacy, outcome expectancy of health and fitness benefits, inter-personal and cultural factors (group work), physical activity monitoring, goal setting, identifying barriers and enablers and health and fitness testing [18]. An expanded explanation of the methodological considerations of the intervention design is available in the paper by Norton and colleagues [18].

The Moderate intervention is designed to involve approximately half the load of the Extensive group. Participants in the Moderate group will attend instructor-led group classes of 60 minutes duration three times per fortnight. Participants will then be required to carry out a minimum of 30 minutes of self-directed activities on a further two days per week. The Moderate exercise intervention is designed to increase MVPA by approximately 150 minutes per week (equivalent to approximately 4000 kJ/week). This dosage is consistent with the minimum level

of energy expenditure recommended for exercise beginners by the United States Surgeon General (600 kJ/day) [19]. Table 1 shows the activities and the progressive increase in planned energy expenditure during the intervention for the Moderate group.

Participants in the Extensive group will attend instructor-led group classes of 60 minutes duration three times per week. Participants will then be required to carry out a minimum of 30 minutes of self-directed activities on a further four days per week. The extensive exercise intervention is designed to increase MVPA by approximately 300 minutes per week (equivalent to approximately 8000 kJ/week). Overall, participants will be required to complete at least 30 minutes of activity every day for 40 days. Table 2 shows the activities and the progressive increase in planned energy expenditure during the intervention for the Extensive group. To reflect the modified nature of the 40-day program in this study for the Moderate exercise group, the intervention conditions will now be referred to as the UniSA Physical Activity (PA) program.

Both intervention groups will be required to keep a physical activity diary and to wear a heart rate monitor for all physical activity sessions greater than 10 minutes (Polar S610i). Participants will be asked to record information for each exercise session including activity type and rating of perceived exertion [20] as well as details form their heart rate monitor including session duration, mean heart rate and caloric expenditure. Participants will be asked to present their heart rate monitors weekly for researchers to download and record the exercise files to ensure appropriate program progression and to monitor adherence. All instructor-led sessions will be approximately one-hour in duration, except where indicated on the schedule (Tables 1 and 2), inclusive of a 10-minute warm up and 10-minute cool down/stretching period. Prior to commencing the intervention, all participants in the intervention groups will attend an introductory information session where they will receive information on the health benefits of physical activity and national physical activity recommendations [3]. Participants will also receive their heart rate monitor, individually programmed according to the manufacturer's recommendations [21], physical activity diaries and instructions on how to use their monitors and complete their diary.

Controls

Participants allocated to the control group will be given no specific instructions regarding their lifestyle. They will be wait-listed to receive the UniSA PA program upon completion of all study assessments.

Outcome measures

All participants will undergo a number of measurements at five time periods during the study: baseline or week zero (before the program begins), mid-program (weeks 3–4), end-program (week 5–6), and at 3 month and 6 month follow-up. These measurements will include doubly labeled water, resting metabolic rate, use of time recalls, accelerometry, various anthropometric and physiological measures and submaximal VO_{2max} tests. Figure 1 provides an overview of the outcome measures assessed at each time point. All outcome measures will be administered by trained, blinded research assistants.

Doubly labeled water

Measurement of total energy expenditure by doubly labeled water will be carried out at baseline and end of the program. Doubly labeled water is considered the gold standard method of energy expenditure measurement in humans [13]. The method is non-invasive and relies on the differential elimination of two stable non-radioactive isotopes, ^{2}H and ^{18}O, which together compose doubly labeled water ($^{2}H_2^{18}O$). While both the ^{2}H and ^{18}O isotopes are lost from the body in the form of water, i.e. from urine, sweat, lung losses and insensible losses, only the ^{18}O isotope is also lost from the body as expired carbon dioxide. The difference in the elimination of these two isotopes is proportional to the carbon dioxide production rate and, therefore, total energy expenditure [13,22,23].

Participants will be administered a dose of doubly labeled water after providing a pre-dosing urine sample. The dose will be administered as 1.55 g per kg of body weight from a stock dose that will combine 1.5 $g.kg^{-1}$ body weight of 10% ^{18}O and 0.05 $g.kg^{-1}$ body weight of ^{2}H, with the stock dose prepared based on a body weight of 300 kg. Participants will be instructed to collect a post-stock dose urine sample 4–6 hours following dosing, as well as daily urine samples for the following 14 days (excluding sampling from the first void of the day). Participants will be asked to store the samples in a domestic freezer until they are returned to the university at the end of the collection period. Samples will be analysed for isotope enrichment using isotope ratio mass spectrometry [24]. This method has a relative accuracy of 1% and within-subject precision of 5-8% [25].

Resting metabolic rate

Resting metabolic rate will be measured using indirect calorimetry via a ventilated hood at baseline and end of the program. Participants will be instructed to be rested and fasted for a minimum of 12 hours prior to the measurement of resting metabolic rate and will be tested while lying down in an environmental chamber with an ambient temperature of 24° C and relative humidity of 60% [26]. After a 15-minute equilibration period, respiratory gas will be collected for 30 minutes using a ventilated hood. Minute ventilation (volume of air

Table 1 Moderate group physical activity itinerary (grey shading indicates group exercise session)

	Sunday	Monday	Tuesday	Wednesday	Thursday	Friday	Saturday
**Week 1 600 kJ/session**					Introductory circuit involving stretching and body awareness activities such as climbing, rolling, balancing, hopping, using a range of different surfaces.	Individual exercise session	Individual exercise session
**Week 2 700 kJ/session**			Training circuit with light hand-held weights, step-ups, stair climbing (one flight), fit ball and core stability exercises.	Individual exercise session	Circuit training including light resistance with dumbbells and medicine ball activities, dynamic stretching activities, introduction to boxing and skipping.		Individual exercise session
**Week 3 800 kJ/session**		Individual exercise session	Resistance class with hand-held dumbbells or light barbells (<10kg), dynamic stretching, resistance routines.		Outdoor circuit training including walking with hand-held weights, stair and hill climbing and introduction to jogging.		Individual exercise session
**Week 4 900 kJ/session**			Circuit training including dynamic stretching activities, resistance activities, spin class, boxing.		Individual exercise session		Kayaking in two-person sit-on-top kayaks ~1.5 hours' duration
**Week 5 1000 kJ/session**		Individual exercise session	Outdoor team games-modified soccer or lacrosse.		Circuit training including dynamic stretching activities, resistance activities, jogging or stair climbing, spin class.		Individual exercise session
**Week 6 1200 kJ/session**	Group bush walk ~1.5-2 hours duration	Individual exercise session			Sand dune and beach activities: walking or jogging through sand hills, team games on beach.		

Table 2 Extensive group physical activity itinerary (grey shading indicates group exercise session)

	Sunday	Monday	Tuesday	Wednesday	Thursday	Friday	Saturday
**Week 1 600 kJ/session**		Introductory circuit involving stretching and body awareness activities such as climbing, rolling, balancing, hopping, using a range of different surfaces.	Individual exercise session	Circuit training including light resistance with dumbbells and medicine ball partner activities, dynamic stretching activities, introduction to step-ups.	Individual exercise session	Training circuit with hand held weights, step-ups, stair climbing (one flight), fit ball and core stability exercises.	Individual exercise session
**Week 2 800 kJ/session**	Individual exercise session	Aerobic activities: warm up, stretching exercises, aerobic activities increasing speed, cool down stretching.	Individual exercise session	Circuit training including light resistance with dumbbells and medicine ball activities, dynamic stretching activities, introduction to boxing and skipping.	Individual exercise session	Resistance class with hand-held dumbbells or light barbells (<10kg), dynamic stretching, resistance routines .	Individual exercise session
**Week 3 1000 kJ/session**	Individual exercise session	Outdoor circuit training including walking with hand-held weights, stair and hill climbing, introduction to jogging.	Individual exercise session	Circuit training including light resistance activities, dynamic stretching activities, introduction to spin class.	Individual exercise session	Aerobics class, warm-up, floor exercises o music, stretching and cool down activities.	Individual exercise session
**Week 4 1200 kJ/session**	Individual exercise session	Outdoor circuit training including walking with hand-held weights or jogging, stair climbing, boxing, skipping.	Individual exercise session	Outdoor team games – modified lacrosse.	Individual exercise session	Circuit training including dynamic stretching activities, resistance activities, jogging or stair climbing, spin class.	Kayaking in two-person sit-on-top kayaks ~1.5 hour duration
**Week 5 1400 kJ/session**	Individual exercise session	Outdoor team games – modified soccer.	Individual exercise session	Circuit training including dynamic stretching activities, resistance activities, jogging or stair climbing, spin class.	Individual exercise session	Circuit training including dynamic stretching activities, resistance activities, jogging or stair climbing, spin class.	Individual exercise session
**Week 6 1600 kJ/session**	Group bush walk ~1.5-2 hours duration	Stretching/recovery class – fitball activities, floor exercises.	Individual exercise session	Sand dune and beach activities: walking or jogging through sand hills, team games on beach.	Individual exercise session	A variety of aerobic activities in teams, touch football, netball, chasing games.	

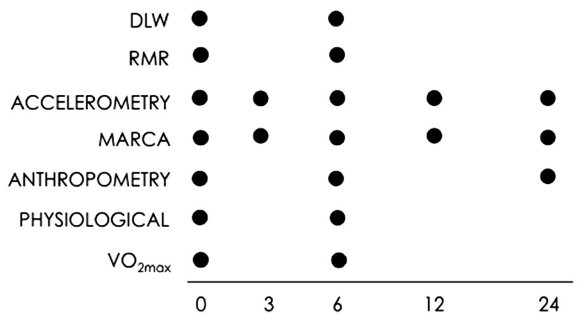

Figure 1 Overview of protocol by outcome measure. MARCA = Multimedia Activity Recall for Children and Adults (use of time recall) RMR = Resting metabolic rate DLW = doubly labeled water.

inhaled and exhaled in one minute), O_2 and CO_2 content will be analysed using a Parvomedics TrueOne 2400 analyser (Parvomedics, Sandy, UT). Resting metabolic rate will be defined as the lowest 5-minute average obtained during the 30-minute measurement period [26]. The TrueOne analyser system has demonstrated reliability and validity, with a within-subject coefficient of variation of 5.4% and comparable results to other validated measures of resting metabolic rate [27].

Accelerometry

Accelerometry will be used to objectively assess the physical activity of participants at all measurement points using the Actigraph GT3X (Actigraph, Penascola, FL). Participants will be required to wear the accelerometer on an elastic waist belt aligned with the right anterior axillary line for seven days at each measurement period at all times (including sleep), except when showering, bathing, swimming or engaging in contact sports. Participants will be asked to complete a brief log to record whether and when they removed the monitor for any reason. Accelerometers will be set to capture 30 second epochs and minimum wear time will be defined as 10 hours per day for four of the seven days, one of which must be a weekend day. Non-wear time will be defined as 60 minutes of consecutive zeros. Total counts and minutes spent in sedentary, light, moderate and vigorous physical activity domains according to the vertical axis will be calculated using the Actlife 5.5 software (Actigraph, Penascola, FL) using cut-offs previously described by Troiano and colleagues [28].

The Actigraph has demonstrated inter-device reliability (r=0.98) [29] and validity against indirect calorimetry when worn on the hip (r=0.56, p<0.001) [30] in assessing activity in adult populations. In a recent comparison of four accelerometers assessing standardised bouts of treadmill walking across multiple trials, the Actigraph was found to have the least variability across accelerometers and the highest overall reliability [31].

Multimedia activity recall for children and adults

Use of time will be measured via telephone interview using the Multimedia Activity Recall for Children and Adults (MARCA). The MARCA is a computerised 24-hour recall tool and will be administered at all measurement points of the study. The MARCA asks participants to recall their previous day from midnight to midnight using meal times as anchor points in a segmented day format. Participants recall their day in time slices of five minutes or more by choosing from 520 different activities organised under a number of categories, such as “Self-Care”, “Occupation” or “Sport/Recreation”. Each activity in the MARCA is assigned a metabolic equivalents (METs) value based on an expanded version of the Ainsworth compendium so that energy expenditure can be estimated [32,33].

Originally designed for use in children and adolescents [34], the MARCA has been modified for use in adults [35]. The adult version of the MARCA has test-retest reliabilities in adults of 0.990-0.997 ($p \leq 0.0001$) for MVPA, physical activity level (PAL; average daily rate of energy expenditure in METs), sleep and screen time, and convergent validity between physical activity level (estimated average rate of energy expenditure) and accelerometer counts/minute of rho = 0.72 [35]. A recent comparison with the gold standard doubly labeled water showed correlations of rho = 0.70 for total daily energy expenditure (Foley et al. 2012).

At each time point, the MARCA will be administered on two occasions, each time recalling two consecutive days, which must include one weekend day and one weekday (such as Friday and Saturday) resulting in a total of four recalled days at each measurement period. For analyses, the four recall days will be averaged using a 5:2 weighting for weekdays: weekend days. Moderate to vigorous physical activity minutes will be calculated as the average number of minutes spent in activities expected to elicit ≥3 METs according to the MARCA compendium. Physical activity level (PAL, in METs) will be calculated using the factorial method, that is by multiplying the rate of energy expenditure associated with each activity (in METs) by the number of minutes for which that activity was performed, summing them across the day, and dividing by 1440 (minutes per day). To determine time spent in zones of energy expenditure, minutes spent in five mutually exclusive and exhaustive energy expenditure zones: 0.0-0.9 METs (sleep); 1.0-1.9 METs (very light physical activity, VLPA); 2.0-2.9 METs (light physical activity, LPA); 3.0-5.9 METs (moderate physical activity, MPA); and ≥6 METs (vigorous physical activity, VPA) will be calculated. Finally, to determine a time use profile, minutes spent in major “activity sets” will be determined, including Physical Activity, Computer, Active Transport, Passive Transport, Quiet Time,

Self-Care, Socio-cultural, Work/Study, Chores, Sleep, and TV/Videogames.

Anthropometry

The following anthropometric measures will be taken at baseline, end-program and at the 24-month follow up: standing height (Leicester Height Measure, Invicta Plastics, Oadby, Leicester, United Kingdom) and mass (Tanita UM-108, Tanita Corporation, China), waist and hip girths, skin fold measurements (triceps, biceps, subscapular and iliac crest) and thee dimensional (3D) whole-body laser scans (Vitus Smart XXL, Human Solutions GmbH, Kaiserslauten, Germany). All physical measurements will be taken according to the International Standards for Anthropometric Assessment [36]. Intra-tester technical error of measurement (TEM) (in a mixed population) is reported as <5.0% for skinfolds and <1.0% for other measures [37]. Inter-tester technical error of measurement (in a mixed population) compared to a criterion anthropometrist is reported as <7.5% for skinfolds and <1.5% for other measures [37]. Measurements extracted from the 3D scans will include girths, as well as segmental and whole body volumes, which will be taken according to the procedures described in Schranz et al. (2010) [38]. Using the hardware-software suite used in this study, scan-extracted girths and volumes have been reported to demonstrate good test-retest reliability (typical error <1.6%) and accuracy (typical errors <1.8%) relative to physical measurements (girths), water displacement plethysmography (segmental volumes) and underwater weighing (whole body volume) [39,40].

Physiological measures

Several physiological measures will be collected at baseline and end-program: blood pressure, total serum cholesterol and fasting blood glucose. Blood pressure will be measured using a non-invasive automated sphygmomanometer (Dinamap Pro 100, GE Medical Systems Information Technologies and Critkon Company, LLC, United Kingdom). Blood pressure will be repeatedly measured until there are two measurements within 5 mmHg for both systolic and diastolic pressure. Total serum cholesterol and fasting blood glucose will be measured using a finger-tip blood sample with automated lancets (Accu-Check Safe-T-Pro Plus, Roche, Australia). Blood specimens will be processed using a Reflotron Plus analyser (Woodley Equipment Company Limited, United Kingdom). Participants will be instructed to be rested and fasted for a minimum of 12 hours prior to the physiological measures.

Cardiorespiratory Fitness

$VO2_{max}$ will be estimated at baseline and end-program using the Physical Work Capacity 75% (PWC75%) sub-maximal bicycle ergometer test [41]. The sub-maximal test will be carried out using an electronically braked cycle ergometer (Ergoselect 200, Ergoline, Germany) and a heart rate monitor and transmitter unit (Polar S160i, Polar, Finland). The test involves 3 x 3-minute stages with progressively increasing resistance, lasting a total of nine minutes (excluding warm-up). The final workload aims to elicit a heart rate that is close to 75% of the participant's age-predicted maximal heart rate (where predicted HR_{max} = 220 minus age of participant). A linear regression equation then allows an estimate of the maximal workload at the participant's predicted maximal heart rate.

Statistical analysis

As this study is aiming to address a mechanism rather than test the effectiveness of the exercise intervention, analysis will be carried out on a per protocol basis where only those participants who complete the intervention will be included in the analyses. Subsequent analyses will also be carried out based on 50% and 75% compliance thresholds with the prescribed volume of physical activity according to objectively monitored adherence (based on heart rate monitoring).

Socio-demographic and anthropometric data will be analysed descriptively. Baseline characteristics of the three groups will be presented but not formally tested for differences. Random effects mixed modelling will be used with time (0, 3, 6, 12 and 24 weeks) and group allocation (*Control, Moderate, Extensive)* as the fixed factors. A time by group interaction term will be used to formally test the differences between the groups. Alpha will be set at 0.05. The response variables for the primary analysis investigating the activitystat hypothesis will be total and activity energy expenditure derived from doubly labeled water, resting metabolic rate derived from indirect calorimetry, total counts and minutes spent in sedentary, light and MVPA derived from accelerometry and minutes spent in MVPA, physical activity superdomain and physical activity level derived from the MARCA.

Response variables for secondary analyses will include use of time derived from the MARCA to determine how participants adjust their daily activity patterns when commencing an exercise program and anthropometric (height, mass, girths, skin folds, volumes) and physiological variables (blood pressure, fasting blood glucose and total serum cholesterol, VO_{2max}) to investigate dose response relationships between physical activity and anthropometric and physiological variables.

A priori power calculations determined that a sample of 26 participants per group (n=78) should be able to detect small to moderate effect sizes (Cohen's d=0.2-0.6) for within groups (time effect) and group x time

interactions, at 5% alpha level and 80% power. Due to documented dropout rates of physical activity interventions [4] and the intensive assessment protocol for this study, a dropout rate of 25% will be anticipated and therefore a total of 46 participants per group (n=138) will be recruited.

Discussion

The possible existence of an activitystat is gaining increasing attention in the physical activity literature. However to date, evidence is largely observational and there has been limited experimental research specifically designed to investigate its existence. To our knowledge, this is the largest experimental, randomised controlled trial to date that has been specifically designed to test the activitystat hypothesis while taking into account the key conceptual and methodological considerations of testing a biologically regulated homeostatic feedback loop.

This study aims to contribute significantly to the physical activity literature by primarily determining the effect of two different volume six-week physical activity interventions in previously inactive adults on total energy expenditure and physical activity. In addition, this study is aiming to comprehensively chart time use patterns in accommodating a new exercise program and dose response relationships between physical activity and anthropometric and physiological outcomes.

Abbreviations

MVPA: Moderate to vigorous physical activity; PA: Physical activity; METs: Metabolic equivalents; PAL: Physical activity level; MARCA: Multimedia activity recall for children and adults; 3D: Three dimensional; PWC75%: Physical work capacity 75%.

Competing interests

The authors declare that they have no competing interests that are directly related to the content of this manuscript.

Authors' contributions

All authors contributed to the protocol design and reviewed and edited the manuscript. SG, TO, CM and CE conceived the idea of the study. TO, KN, JD and AE are Chief Investigators on NHMRC Project Grant #631916. SG drafted the manuscript, and contributed to the doubly-labeled water protocol. KN and NL devised the 40-day fitness program protocol. JD contributed to the accelerometry protocol. GT contributed to the anthropmetry protocol. AE contributed to the statistical analysis. TO authored the MARCA software and devised the use of time analysis. All authors read and approved the final manuscript.

Authors' information

SG is a PhD student with the School of Health Sciences and a member of the Health and Use of Time (HUT) Group. CM is an Australian Research Council Post Doctoral Award Fellow, based in the Health and Use of Time (HUT) Group, within the School of Health Sciences at the University of South Australia. KN is a Professor of Exercise Science at the University of South Australia. JD is a senior lecturer in exercise science at the University of South Australia, and co-leader of the Exercise for Health and Human Performance (EHHP) research group. GT is a Senior Lecturer in the School of Health Sciences at the University of South Australia, and a member of the Health and Use of Time (HUT) Group. AE is Professor of Biostatics at the University of South Australia, and holds the Foundation Chair in that subject. CE is a lecturer at the University of South Australia and is a member of the International Centre for Allied Health Evidence, School of Health Sciences. NL is a researcher in the School of Health Sciences at the University of South Australia, and part of the Exercise for Health and Human Performance (EHHP) Research Group. TO is Professor of Health Sciences at the University of South Australia, and Director of the Health and Use of Time (HUT) Group.

Acknowledgments

This study is funded by a National Health and Medical Research Council Project Grant (#631916). Ms Gomersall is supported by an Australian Postgraduate Award Scholarship. Dr English is supported by a National Health and Medical Research Council Training Fellowship (#610312). Dr Maher is supported by an Australian Research Council post-doctoral fellowship. Dr Dollman is supported by a South Australian Cardiovascular Research Development Program Fellowship.

Author details

[1]Health and Use of Time (HUT) Group, Sansom Institute for Health Research, University of South Australia, Adelaide, South Australia, Australia. [2]Exercise for Health and Human Performance Group, Sansom Institute for Health Research, University of South Australia, Adelaide, South Australia, Australia. [3]School of Nursing and Midwifery, University of South Australia, Adelaide South, Australia, Australia. [4]International Centre for Allied Health Evidence (iCAHE), School of Health Sciences, University of South Australia, Adelaide, South Australia, Australia.

Received: 4 August 2012 Accepted: 3 October 2012
Published: 8 October 2012

References

1. Department of Health, Physical Activity, Health Improvement and Protection: *Start Active, Stay Active: A report on physical activity from the four home countries' Chief Medical Officers*. London, United Kingdom; 2011.
2. U.S. Department of Health and Human Services: *2008 Physical Activity Guidelines for Americans*. United States of America; 2008.
3. Department of Health and Aged Care: *National Physical Activity Guidelines for Australians*. Canberra: Department of Health and Aged Care; 1999.
4. Foster C, Hillsdon M, Thorogood M: **Interventions for promoting physical activity.** *Cochrane Database of Systematic Reviews* 2005, (1). doi:10.1002/14651858.CD003180.pub2. Art. No.: CD003180.
5. Begg S, Vos T, Barker B, Stevenson C, Stanley L, Lopez A: *The burden of disease and injury in Australia 2003*. Canberra: PHE82, Australian Institute for Health and Welfare; 2003.
6. Australian Institute for Health an Welfare: *Australia's Health 2010*. Canberra: 2010.
7. Rowland TW: **The biological basis of physical activity.** *Med Sci Sports Exerc* 1998, **30**(3):392.
8. Eisenmann JC, Wickel EE: **The biological basis of physical activity in children: revisited.** *Pediatr Exerc Sci* 2009, **21**(3):257–272.
9. Garland T Jr, Schutz H, Chappell MA, Keeney BK, Meek TH, Copes LE, Acosta W, Drenowatz C, Maciel RC, Van Dijk G, *et al*: **The biological control of voluntary exercise, spontaneous physical activity and daily energy expenditure in relation to obesity: Human and rodent perspectives.** *J Exp Biol* 2011, **214**(2):206–229.
10. Bouchard C, Rankinen T: **Are people physically active because of their genes.** *Pres Council Phys Fit* 2006, **7**:1–8.
11. Thorburn A, Proietto J: **Biological determinants of spontaneous physical activity.** *Obes Rev* 2000, **1**(2):87–94.
12. Gomersall S, Rowlands A, English C, Maher C, Olds T: **The ActivityStat hypothesis: the concept, the evidence, and the methodologies.** *Sports Med* 2012, *In press*. Accepted 9 July 2012.
13. Bluck LJC: **Doubly labelled water for the measurement of total energy ependiture in man - progress and applications in the last decade.** *Nurtrition Bulletin* 2008, **33**:80–90.
14. Sallis JF: **Age-related decline in physical activity: a synthesis of human and animal studies.** *Med Sci Sports Exerc* 2000, **32**(9):1598–1600.
15. Tucker P, Gilliland J: **The effect of season and weather on physical activity: A systematic review.** *Public Health* 2007, **121**:909–922.
16. Australian Institute for Health and Welfare: *The Active Australia Survey: a guide and manual for implementation, analysis and reporting*. Canberra; 2003.

17. Sports Medicine Australia: *Sports Medicine Australia pre-exercise screening system*. Canberra: Commonwealth Department of Health and Ageing; 2005.
18. Norton L, Norton K, Lewis N, Dollman J: **A comparison of two short-term intensive physical activity interventions: methodological considerations.** *Int J Behav Nutr Phys Act* 2011, **8**:133.
19. Services UDoHaH: *Physial activity and health: a report of the surgeon general Atlanta*. GA: Centres for Disease Control and Prevention, National Centre for Chronic Disease Prevention and Health Promotion; 1996.
20. Borg GAV: **Psychophysical bases of percieved exertion.** *Med Sci Sports Exerc* 1982, **14**(5):377–381.
21. Electro™ P: *S610™ Heart Rate monitor User's Manual*. Kempele, Finland: Polar Electro Inc; 2004.
22. Schoeller DA: **Energy expenditure from doubly labeled water: some fundamental considerations in humans.** *Am J Clin Nutr* 1983, **38**:999–1005.
23. Ainslie PN, Reilly T, Westerterp KR: **Estimating human energy expenditure.** *Sports Med* 2003, **9**(33):683–698.
24. Speakman JR: *Doubly labelled water: Theory and practice*. Great Britian: University Press, Cambridge; 1997.
25. Schoeller DA: **Validation of habitual energy intake.** *Public Health Nurtition* 2002, **6A**(6):883–888.
26. Compher C, Frankenfield D, Keim N, Roth-Yousey L: **Best practive methods to apply to measurement of resting metabolic rate in adults: a systematic review.** *J Am Diet Assoc* 2006, **106:**881–903.
27. Cooper JA, Watras AC, O'Brien MJ, Luke A, Dobratz B JR, Earthman CP, Schoeller DA: **Assessing validity and reliability of resting metabolic rate in six gas analysis systems.** *J Am Diet Assoc* 2009, **109:**128–132.
28. Troiano R, Berrigan D, Dodd KW, Mâsse LC, Tilert T, McDowell M: **Physical activity in the United States measured by accelerometer.** *Med Sci Sports Exerc* 2008, **40**(1):181–188.
29. Brage S, Wedderkopp N, Franks PW, Andersen LB, Froberg K: **Re-examination of validity and reliability of the CSA monitor in walking and runnin.** *Med Sci Sports Exerc.* 2003, **35**(8):1447–1454.
30. Swartz AM, Strath SJ, Bassett DR, O'Brien WL, King GA, Ainsworth BE: **Estimation of energy expenditure using CSA accelerometers at hip and wrist sites.** *Med Sci Sports Exerc* 2000, **39**(9):S450–S456.
31. Welk GJ, Schaben JA, Morrow JR: **Reliability of accelerometry-based activity monitors: a generalisability study.** *Med Sci Sports Exerc* 2004, **36**(9):1637–1645.
32. Ainsworth BE, Haskell WL, Herrmann SD, Meckes N, Bassett DRJ, Tudor-Locke C, Greer JL, Vezina J, Whitt-Glover MC, Leon AS: **Compendium of physical activities: a second update of codes and MET values.** *Med Sci Sports Exerc* 2011, **43**(8):1575–1581.
33. Ainsworth BE, Haskell WL, Whitt MC, Irwin ML, Swartz AM, Strath SJ, O'Brien WL, Bassett DRJ, Schmitz KH, Emplaincourt PO, *et al*: **Compendium of physical activities: an update of activity codes and MET intensities.** *Med Sci Sports Exerc* 2000, **32**(9):S498–S516.
34. Ridley K, Olds TS, Hill A: **The multimedia activity recall for children and adolescents (MARCA): development and evaluation.** *Int J Behav Nutr Phys Act* 2006, **3**(10). doi:doi:10.1186/1479-5868-3-10.
35. Gomersall S, Olds TS, Ridley K: **Development and evaluation of an adult use-of-time instrument with an energy expenditure focus.** *J Sci Med Sport* 2011, **14**(2):143–148.
36. Marfell-Jones M, Olds T, Stewart A, Carter L: *International standards for anthropometric assessment*. Potchefstroom, South Africa: International Society for the Advancement of Kinanthropometry; 2006.
37. Gore C, Norton K, Olds T, Whittingham N, Birchall K, Clough M, Dickerson B, Downie I: **Accreditation in anthropometry: an Australian model.** In *Anthropometrica*. Edited by Norton K, Olds TS. Sydney, New South Wales: University of new South Wales Press; 1996:395–411.
38. Schranz N, Tomkinson G, Olds T, Daniell N: **Three-dimensional anthropometric analysis: differences between elite Australian rowers and the general population.** *J Sports Sci* 2010, **28**:459–469.
39. Daniell N: **A comparison of the accuracy of the Vitus smart and Hammatsu body line 3D whoe-body scanners.** In *Kinanthropometry X*. Edited by Mafell-Jones M, Olds T. London, New York: Routledge; 2008:39.
40. Collins J: *Volumetric analysis of human bodies*. Australia: Unpublished honours thesis, University of South Australia; 2006.
41. Gore C, Booth M, Bauman A, Owen N: **Utility of pwc75% as an estimate of aerobic power in epidemiological and population based studies.** *Med Sci Sports Exerc* 1999, **31**(2):348–351.

doi:10.1186/1471-2458-12-851
Cite this article as: Gomersall *et al.*: Testing the activitystat hypothesis: a randomised controlled trial protocol. *BMC Public Health* 2012 **12**:851.

Submit your next manuscript to BioMed Central and take full advantage of:

- **Convenient online submission**
- **Thorough peer review**
- **No space constraints or color figure charges**
- **Immediate publication on acceptance**
- **Inclusion in PubMed, CAS, Scopus and Google Scholar**
- **Research which is freely available for redistribution**

Submit your manuscript at
www.biomedcentral.com/submit

BioMed Central
