# Supplementary material for: Time Regained: When People Stop a Physical Activity Program, How Does Their Time Use Change? A Randomised Controlled Trial
Source: PLoS One. 2015 May 29;10(5):e0126665. doi: 10.1371/journal.pone.0126665 (PMC4449013; doi:10.1371/journal.pone.0126665)
Supplement: S1 Table — Note: Summary data are raw scores and significant differences are indicated in bold. Sample includes intervention participants only sub-divided into low compliers (attended <70% of prescribed physical activity program) and high compliers (attended ≥70% of prescribed physical activity program). N = 73 (Low compliers, n = 37, High compliers n = 36). SD = standard deviation, TV = television. (PDF) [file pone.0126665.s003.pdf]

Table S1. Time (min/day) spent in each time use superdomain in low compliers compared with high compliers, measured by the MARCA.

Superdomain	Period	Low compliers Mean (SD)	High compliers Mean (SD)	P Compliance x Time
**TV / Videogames**	**Baseline**	120 (72)	132 (86)	0.99
	**Mid**	113 (73)	92 (60)	0.23
	**End**	99 (61)	97 (73)	0.37
	**3 month**	100 (63)	117 (78)	0.42
	**6 month**	108 (61)	113 (76)	0.78
**Computer**	**Baseline**	191 (110)	178 (108)	0.67
	**Mid**	166 (127)	145 (120)	0.58
	**End**	152 (114)	133 (88)	0.33
	**3 month**	178 (133)	182 (154)	0.42
	**6 month**	181 (115)	174 (124)	0.48
**Active Transport**	**Baseline**	63 (45)	54 (30)	0.28
	**Mid**	62 (36)	71 (37)	0.39
	**End**	63 (30)	72 (31)	0.39
	**3 month**	55 (30)	72 (47)	0.08
	**6 month**	55 (35)	51 (28)	0.58
**Passive Transport**	**Baseline**	76 (35)	81 (44)	0.68

	**Mid**	78 (39)	86 (38)	0.33
	**End**	87 (45)	87 (37)	0.81
	**3 month**	82 (44)	82 (41)	0.65
	**6 month**	82 (50)	82 (45)	0.76
**Chores**	**Baseline**	117 (75)	139 (97)	0.23
	**Mid**	117 (53)	118 (84)	0.80
	**End**	109 (59)	139 (89)	0.15
	**3 month**	119 (59)	135 (98)	0.31
	**6 month**	122 (65)	127 (83)	0.99
**Sleep**	**Baseline**	508 (77)	480 (73)	0.12
	**Mid**	530 (71)	488 (57)	**0.03**
	**End**	513 (85)	492 (64)	0.31
	**3 month**	507 (83)	485 (68)	0.26
	**6 month**	506 (62)	508 (124)	0.93
**Physical Activity**	**Baseline**	14 (27)	6 (10)	**0.05**
	**Mid**	16 (19)	43 (23)	**<0.01**
	**End**	36 (47)	43 (30)	0.26
	**3 month**	17 (29)	17 (28)	0.97
	**6 month**	14 (27)	10 (16)	0.96
**Quiet Time**	**Baseline**	54 (45)	56 (43)	0.5
	**Mid**	42 (42)	53 (36)	0.08

	**End**	53 (50)	56 (55)	0.48
	**3 month**	54 (54)	48 (34)	0.55
	**6 month**	55(51)	59 (44)	0.28
**Self-Care**	**Baseline**	111 (31)	123 (34)	0.13
	**Mid**	124 (41)	123 (31)	0.90
	**End**	120 (31)	132 (38)	0.15
	**3 month**	127 (34)	129 (40)	0.75
	**6 month**	119 (30)	123 (38)	0.60
**Work & Study**	**Baseline**	65 (80)	82 (77)	0.45
	**Mid**	78 (84)	110 (110)	0.31
	**End**	97 (104)	86 (81)	0.53
	**3 month**	107 (127)	81 (86)	0.73
	**6 month**	74 (81)	83 (77)	0.90
**Socio-Cultural**	**Baseline**	123 (82)	110 (66)	0.52
	**Mid**	114 (83)	110 (71)	0.59
	**End**	108 (70)	101 (51)	0.89
	**3 month**	105 (89)	93 (69)	0.62
	**6 month**	124 (74)	110 (77)	0.49

Note: Summary data are raw scores and significant differences are indicated in bold. Sample includes intervention participants only sub-divided into low compliers (attended <70% of prescribed physical activity program) and high compliers (attended ≥70% of prescribed

physical activity program). N =73 (Low compliers, n=37, High compliers n=36).
SD=standard deviation, TV=television.
